# The transcriptional regulator CueR positively regulates the expression of the *cueRA* operon to maintain copper homeostasis in *Pseudomonas chlororaphis* zm-1

**DOI:** 10.1128/spectrum.03981-25

**Published:** 2026-09-17

**Authors:** Zhongkang Wang, Chen Li, Zhenming Lei, Sijia Duan, Fengying Liu, Shaowei Wang, Gang Wang

**Affiliations:** 1 School of Life Sciences, Henan University 12411, Kaifeng, China; 2 Engineering Research Center for Applied Microbiology of Henan Province, Kaifeng, China; Luonnonvarakeskus, Oulu, Finland; Albert-Ludwigs-Universitat Freiburg, Freiburg, Germany

**Keywords:** *Pseudomonas chlororaphis*, copper homeostasis, *cueRA *operon, rhizosphere colonization

## Abstract

Copper serves as an indispensable trace element in living organisms, playing pivotal roles in fundamental physiological processes including enzyme assembly, antioxidant defense, and metabolic regulation. However, excess copper can induce oxidative damage. MerR-family regulator CueR acts as the core mediator of copper homeostasis in prokaryotes, but its mechanism of action in *P. chlororaphis* zm-1, a plant biocontrol bacterium that inhibits peanut stem rot, remains unclear. In this study, we investigated the regulatory function of CueR using molecular techniques including gene deletion, electrophoretic mobility shift assay, and quantitative real-time PCR. The results demonstrated that the transcriptional regulator gene *cueR* and the P_1B_-type ATPase gene *cueA* (involved in copper efflux) form the *cueRA* operon in strain zm-1, with a 4-bp overlap and co-transcriptional feature. CueR directly binds to the promoter region and positively activates *cueRA* expression upon copper ion induction, thereby driving CueA-mediated efflux of excess copper ions. The deletion of *cueR* or *cueA* significantly reduced copper tolerance, increased intracellular copper content, and impaired peanut rhizosphere colonization ability of the strain, while these phenotypes were restored in the complementation strains. This study deciphers the regulatory mechanism of the *cueRA* operon in *P. chlororaphis* and establishes a correlation analysis between this mechanism and the rhizosphere colonization capacity of the strain. The findings provide a molecular basis for understanding the diversity of copper homeostasis regulation in *Pseudomonas* and optimizing the biocontrol application of this bacterium.

**IMPORTANCE:**

The CueR-regulated copper efflux system is essential for bacterial survival under copper overload conditions. While this system has been extensively characterized in human pathogens, its function in plant-beneficial bacteria remains poorly understood. Here, we investigate the agricultural biocontrol strain *P. chlororaphis* zm-1 and delineate the regulatory mechanism of CueR through molecular approaches, leading to the identification of the *cueRA* operon. Furthermore, we establish a functional link between CueR and rhizosphere colonization, thereby providing a framework for understanding CueR-mediated regulation in plant-associated bacteria.

## INTRODUCTION

As an essential trace metal element, copper exists in nearly all life forms ranging from bacteria to mammals (1, 2). Bioinformatics analyses have revealed that the size of the copper proteome in eukaryotes and prokaryotes is usually less than 1% of the total organismal proteome (3). Nevertheless, these copper-binding proteins play pivotal roles in numerous biochemical and physiological processes. Among them, copper-containing enzymes such as cytochrome *c* oxidase, peroxidase, and copper/zinc—superoxide dismutase perform crucial functions in cellular energy metabolism, redox reactions, and defense against oxidative stress (4). However, excess copper is highly toxic. Excess Cu²^+^ disrupts the intracellular reductive environment, while Cu^+^ reacts with hydrogen peroxide via the Fenton reaction to generate harmful hydroxyl radicals (5). These hydroxyl radicals further produce more reactive oxygen species through the Haber-Weiss cycle, thereby damaging the cytoplasmic membrane, nucleic acids, and lipids (6, 7). More critically, excess Cu^+^ displaces Fe²^+^ in the iron-sulfur clusters of dehydrogenases, leading to the inactivation of key enzymes including dihydroxy-acid dehydratase, isopropylmalate isomerase, fumarase A, and 6-phosphogluconate dehydratase (8–10), which ultimately results in cellular dysfunction or even death.

Given this double-edged sword effect of copper, its homeostasis in organisms requires strict regulation. Reported studies have demonstrated that P-type ATPases play a major role in copper detoxification in organisms. As a large superfamily of proteins widely distributed in various organisms, members of this family hydrolyze ATP to obtain energy for transmembrane transport of substrates (11). P-type ATPases share a common topological structure, consisting of three cytoplasmic domains (actuator, phosphorylation, and nucleotide-binding domains) and a transmembrane domain composed of at least six transmembrane helices (12). Based on sequence homology, the P-type ATPase family is divided into 5 branches (Types I–V), which are further classified into 10 distinct subtypes or classes according to substrate specificity (11, 13). P_1B_-type ATPases, also known as CPx-type ATPases, are defined by the conserved intramembrane CPx motif (Cys-Pro-Cys/His) (14). Members of this subgroup include CopA, ZntA, and CadA (15–18), which are responsible for the transport of “soft” transition metal ions such as copper, zinc, lead, and cobalt in bacteria (19). Among them, CopA primarily mediates copper transport (20, 21).

In Gram-negative bacteria such as *E. coli*, CopA is regulated by CueR to maintain copper homeostasis (22, 23). As a member of the MerR family, CueR functions as a dimer, exerting both activating and repressive effects at the same site between the −35 and −10 regions of the promoter (24). When intracellular copper ion levels are below the threshold, CueR binds to the promoter region via its N-terminal helix-turn-helix (HTH) DNA-binding domain (25). Upon elevated copper ion levels, CueR recognizes Cu^+^ through a high-affinity coordination bond formed by two conserved cysteine residues in its C-terminal domain and is activated at subnanomolar Cu^+^ concentrations. The binding of Cu^+^ triggers a conformational change in the CueR dimer, which distorts the DNA to enable the correct binding of σ^70^-type RNA polymerase and initiate transcription (26, 27).

Currently, the regulatory mechanism of CueR on P_1B_-type ATPases (CopA/CueA) has been reported in human and mammalian pathogenic bacteria such as *E. coli*, *Vibrio cholerae*, and *Vibrio parahaemolyticus* (22, 23, 28) but remains less elaborated in plant growth-promoting rhizobacteria (PGPR). Notably, the precise regulation of copper homeostasis in plant root-associated PGPR is closely associated with their functional performance in ecosystems. *Pseudomonas*, as an important group of PGPR, has been widely recognized for its functions in agricultural ecosystems, including biological control, biofertilization, and plant stimulation (29, 30). These strains can effectively inhibit agricultural harmful microorganisms such as pathogenic fungi, pathogenic bacteria, and nematodes through active substances including secondary metabolites, siderophores, and volatile organic compounds (31–34). Meanwhile, they can promote plant growth and induce plant immune responses, thus being frequently developed as microbial inoculants for agricultural production (32, 35).

Previously, our research group isolated and identified a biocontrol strain *P. chlororaphis* zm-1. Studies have shown that phenazine-1-carboxylic acid, a secondary metabolite of this strain, exhibits strong inhibitory activity against *Sclerotium rolfsii*—the pathogenic fungus causing peanut stem rot (36). Given the important biocontrol value of *P. chlororaphis* zm-1 and the critical impact of copper homeostasis on its colonization ability, systematic elucidation of the regulatory mechanism of its copper homeostasis system holds significant scientific significance. Here, we focused on *P. chlororaphis* zm-1 to investigate the regulatory relationship between CueR and the copper transporter gene *cueA*. We clarified the molecular characteristics of their co-expression as an operon and elucidated the specific concentration threshold of this system in response to copper stress. This research provides an important theoretical basis for understanding the regulation of copper homeostasis in *Pseudomonas* and lays a foundation for further optimizing the biocontrol application of this strain.

## RESULTS

### *P. chlororaphis* zm-1 harbors a copper-responsive transcriptional regulator CueR and a P_1B_-type ATPase CueA

**Fig 1:**
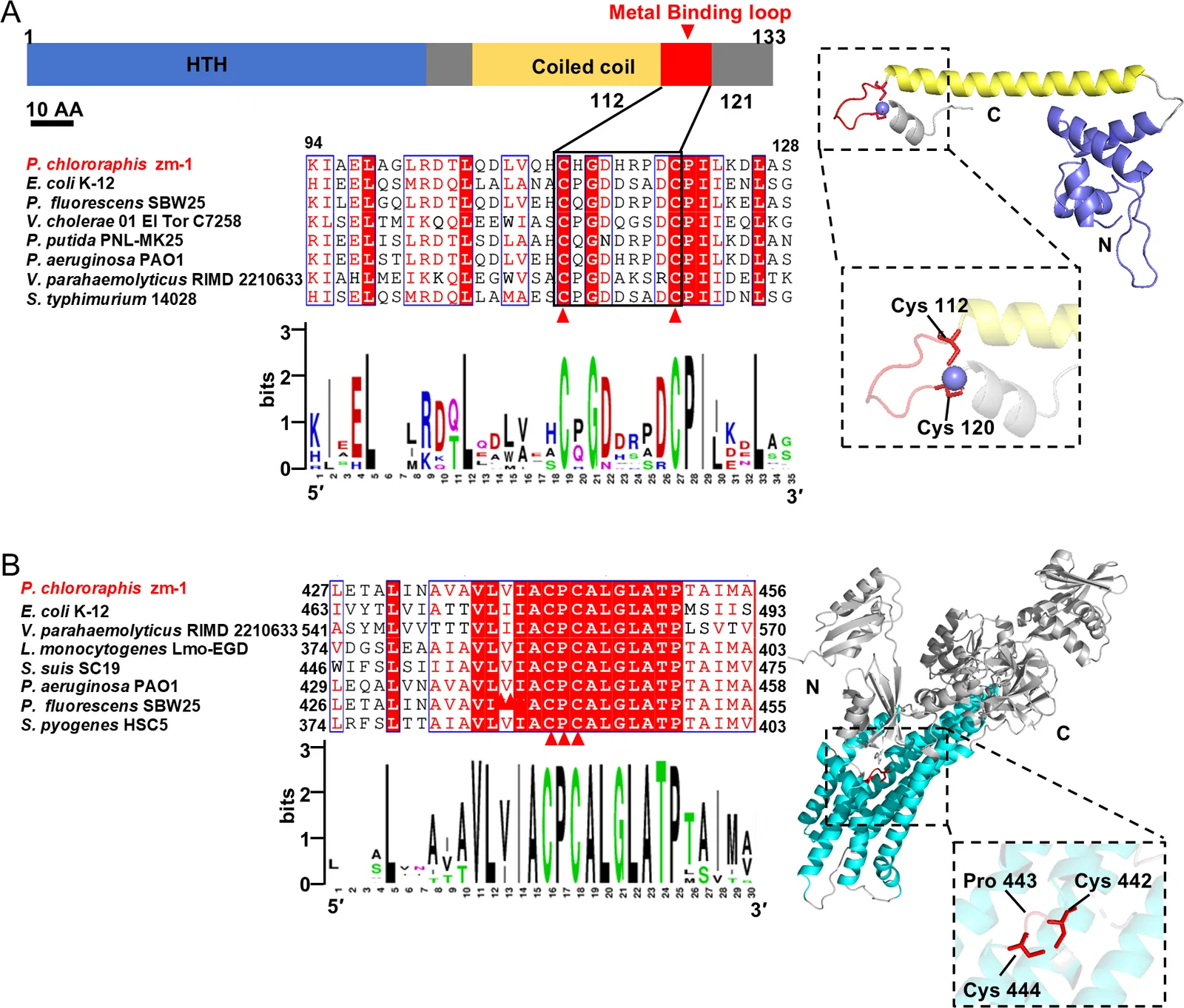
Bioinformatics analysis of CueR and CueA in *Pseudomonas chlororaphis* zm-1**.** (**A**) Domain architecture and conserved motif analysis of CueR. CueR contains three domains: an HTH domain, a coiled-coil domain, and a metal-binding loop. The metal-binding loop spans residues 112–121, with Cys residues at positions 112 and 121 responsible for Cu^+^ coordination. Multiple sequence alignment of the metal-binding loop domain of CueR from *P. chlororaphis* zm-1 with those of reported CueR proteins from other species, including *E. coli*, *Pseudomonas fluorescens*, *Vibrio cholerae*, *Pseudomonas putida*, *Pseudomonas aeruginosa*, *Vibrio parahaemolyticus*, and *Salmonella typhimurium*, is shown. The region enclosed by the black box indicates the metal-binding loop in each species. The consensus sequence logo of CueR from the different species is displayed below the alignment. The CueR structural model was generated using AlphaFold and visualized with PyMOL. In the structure, the blue region represents the HTH domain, the yellow region represents the coiled-coil domain, and the red region represents the metal-binding loop. The Cu^+^ is depicted as a blue sphere. (**B**) Conserved motif analysis of CueA. Multiple sequence alignment of the conserved amino acid sequence of CueA with those of reported P_1B_-type ATPases from other species is shown. The consensus sequence logo of P_1B_-type ATPases from the different species is displayed below the alignment. The red triangle indicates the core characteristic Cys-Pro-Cys motif. The predicted structural model of CueA was generated using AlphaFold and visualized with PyMOL. The cyan regions represent the transmembrane domains of CueA. The inset highlights the core Cys-Pro-Cys motif.

CueR contains three domains: an H-T-H domain, a central Coiled coil domain, and a C-terminal metal-binding domain. Two conserved cysteine residues in the metal-binding domain are responsible for Cu^+^ binding. Based on the conserved domain characteristics of CueR and previous studies on CueR in *Pseudomonas*, a genome-wide search of *P. chlororaphis* zm-1 was performed, leading to the identification of a CueR homolog encoded by the gene GSF17_RS03490, which was tentatively named CueR. Bioinformatics analysis revealed that this gene has an open reading frame (ORF) of 402 bp, encoding 133 amino acid residues with a predicted molecular weight of approximately 14.8 kDa. Further analysis showed that the N-terminal 1–70 amino acid region of this CueR is an H-T-H domain responsible for DNA binding, while the C-terminal 112–120 amino acid region is a metal-binding domain with highly conserved Cys112 and Cys120 residues involved in Cu^+^ binding (Fig. 1A). Phylogenetic tree analysis indicated that CueR from *P. chlororaphis* zm-1 clusters in the same evolutionary branch as selected homologous CueR proteins (Fig. S1A). It shares 89% sequence identity and 94% sequence similarity with CueR from *Pseudomonas fluorescens* SBW25, 59% identity and 72% similarity with that from *Rhodobacter sphaeroides* ATH 2.4.1, 44% identity and 62% similarity with that from *E. coli* K-12, 43% identity and 60% similarity with that from *Vibrio cholerae* 01 El Tor C7258. This indicates that CueR from *P. chlororaphis* zm-1 has a close genetic relationship and functional similarity with previously reported CueR homologs.

Upstream of *cueR*, a gene annotated as a “heavy metal translocating P-type ATPase” (GSF17_RS03495) was identified. This gene has an ORF of 2,397 bp, overlapping with *cueR* by 4 bp, and encodes 799 amino acid residues with a predicted molecular weight of 83.7 kDa. AlphaFold modeling and amino acid sequence homology alignment showed that it contains the conserved Cys-Pro-Cys (CPC) motif at positions 442–444, a signature of P_1B_-type ATPases (Fig. 1B), and was tentatively named CueA. Phylogenetic tree analysis (Fig. S1B) and multiple sequence alignment revealed that CueA from *P. chlororaphis* zm-1 shares 88% sequence identity and 92% sequence similarity with CueA from *P. fluorescens* SBW25, 47% identity and 68% similarity with CopA from *Listeria monocytogenes* Lmo-EGD, and 39% identity and 56% similarity with CopA from *E. coli* K-12. This indicates that the CueA in *P. chlororaphis* zm-1 is a homolog of CopA, a P_1B_-type ATPase responsible for copper efflux.

### CueR and CueA collectively maintain copper homeostasis in *P. chlororaphis* zm-1

**Fig 2:**
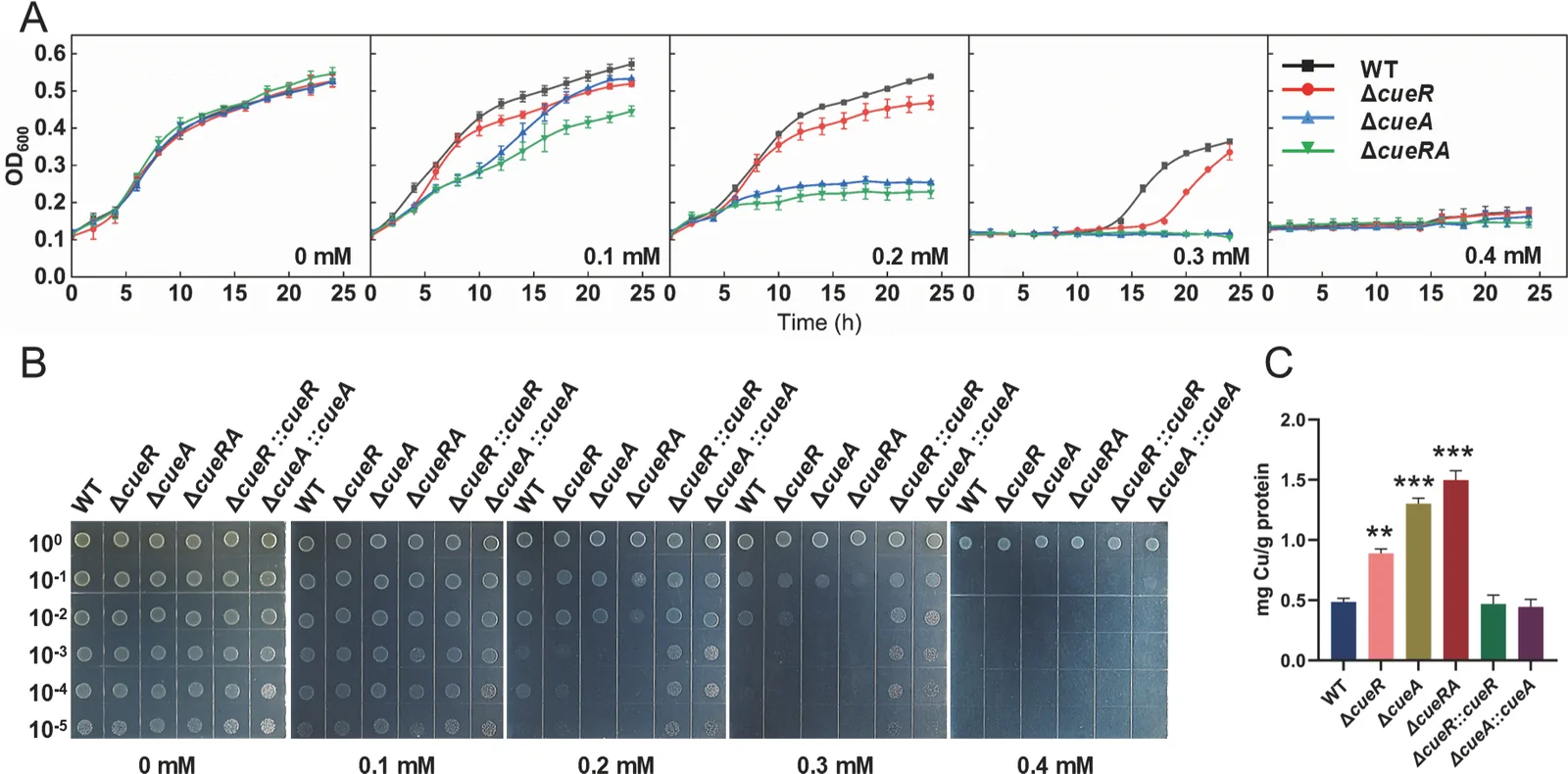
Intracellular copper accumulation impairs growth of mutant strains. (**A**) Growth curves of mutant strains in LB medium supplemented with 0–0.4 mM CuSO₄·5H₂O. (**B**) Spot dilution assays of mutant strains on LB agar plates containing 0–0.4 mM CuSO₄·5H₂O. (**C**) Intracellular copper content measured by atomic absorption spectroscopy. Intracellular copper levels in exponential-phase cells after 30 min exposure to 0.2 mM CuSO₄·5H₂O in LB medium. Data are expressed as mean ± SD (*n* = 3). Two-tailed unpaired Student’s *t*-test was used to calculate *P* values. **P < 0.01, ***P < 0.001.

To investigate the roles of CueR and CueA in maintaining copper homeostasis in *P. chlororaphis* zm-1, we constructed single deletion mutants Δ*cueR* and Δ*cueA*, double deletion mutant Δ*cueRA* (Fig. S2C), and complementation strains Δ*cueR::cueR* and Δ*cueA::cueA* (Fig. S3C). Bacterial growth was assessed in liquid LB containing 0.1–0.4 mM CuSO₄·5H₂O; simultaneously, survival was analyzed via spot dilution assays on LB agar plates supplemented with identical copper concentrations. In liquid LB (Fig. 2A), growth differences among the strains emerged at 0.1 mM CuSO₄·5H₂O and became more pronounced with increasing CuSO₄·5H₂O concentrations. Bacterial growth was completely inhibited at 0.4 mM CuSO₄·5H₂O. Spot dilution assays (Fig. 2B) showed no marked differences in survival among all strains at 0–0.1 mM CuSO₄·5H₂O. However, at 0.2 mM, the Δ*cueA* and Δ*cueRA* mutants formed fewer colonies than the wild-type (WT) and Δ*cueR* strains. When the concentration was raised to 0.3 mM, colony counts of Δ*cueA* and Δ*cueRA* further decreased, and the Δ*cueR* mutant also began to show reduced survival compared to the WT. The copper tolerance of the complementation strains was restored to WT levels. Based on these growth data, we determined the copper tolerance hierarchy of the strains as follows: WT > Δ*cueR* > Δ*cueA* > Δ*cueRA*. To confirm that the observed growth differences were associated with copper accumulation, exponentially growing cells (OD₆₀₀ = 0.5) in liquid LB medium were challenged with 0.2 mM CuSO₄·5H₂O for 2 h. Bacterial cells were then collected and digested with nitric acid, and intracellular copper levels were measured. The results (Fig. 2C) show that the intracellular copper concentrations in the Δ*cueR*, Δ*cueA*, and Δ*cueRA* mutants reached 0.88, 1.30, and 1.50 mg/g dry cell, respectively, which was significantly elevated compared to the WT strain (0.48 mg/g dry cell). In the complementation strains (Δ*cueR::cueR* and Δ*cueA::cueA*), copper accumulation returned to WT levels (0.46 and 0.44 mg/g dry cell).

### CueR regulates *cueA* transcription under copper stress

**Fig 3:**
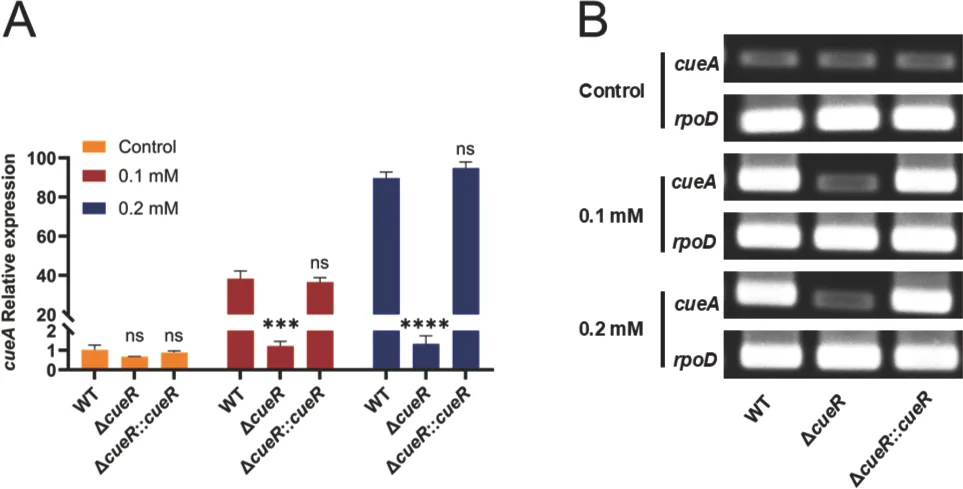
*cueR* is required for copper stress-induced upregulation of *cueA* expression. (**A**) Relative expression levels of *cueA* in the WT strain under 0, 0.1, and 0.2 mM CuSO₄·5H₂O stress. (**B**) Agarose gel electrophoresis of qRT-PCR products. Data are presented as mean ± SD (*n* = 3). Two-tailed unpaired Student’s *t*-test was used to calculate *P* values. ***P < 0.001, ****P < 0.0001, ns, not significant.

To analyze the roles of CueR and CueA in bacterial copper tolerance at the transcriptional level, quantitative real-time PCR (qRT-PCR) was used to quantify *cueA* expression in WT and Δ*cueR* strains treated with 0, 0.1, and 0.2 mM CuSO₄·5H₂O (Fig. 3A and B). In the control group treated with deionized water, there was no significant difference in *cueA* expression between WT and Δ*cueR*. After treatment with 0.1 mM CuSO₄·5H₂O, *cueA* expression in WT and Δ*cueR::cueR* was upregulated by 37-fold and 35-fold compared to the control, respectively, while *cueA* expression in Δ*cueR* only increased by 0.2-fold. When the CuSO₄·5H₂O concentration was increased to 0.2 mM, *cueA* expression in WT and Δ*cueR::cueR* was upregulated by 89-fold and 94-fold compared to the control, respectively, whereas *cueA* expression in Δ*cueR* only increased by 0.3-fold. These results indicate that *cueA* is involved in copper tolerance in *P. chlororaphis* zm-1, with its expression increasing with copper concentration. Furthermore, the deletion of *cueR* blocks *cueA* upregulation under copper stress, demonstrating that CueR is required for *cueA* expression under copper stress.

### Cu^+^ enhances the binding of CueR to the *PcueA* region

To further verify the regulatory relationship between CueR and *cueA*, the upstream sequence of the *cueA* gene was analyzed to identify its promoter region. Alignment with the reported CueR-binding region in *E. coli* revealed that the *cueA* promoter (*PcueA*) contains a palindromic consensus motif (5′-ACCTTGCCATGCTGGCAAGGT-3′) recognized by CueR, suggesting that CueR directly binds to the *PcueA* region in *P. chlororaphis* zm-1. To demonstrate this direct binding ability, we expressed and purified the CueR protein (Fig. S4), and in parallel, obtained fluorescently labeled *PcueA* fragment by PCR.

**Fig 4:**
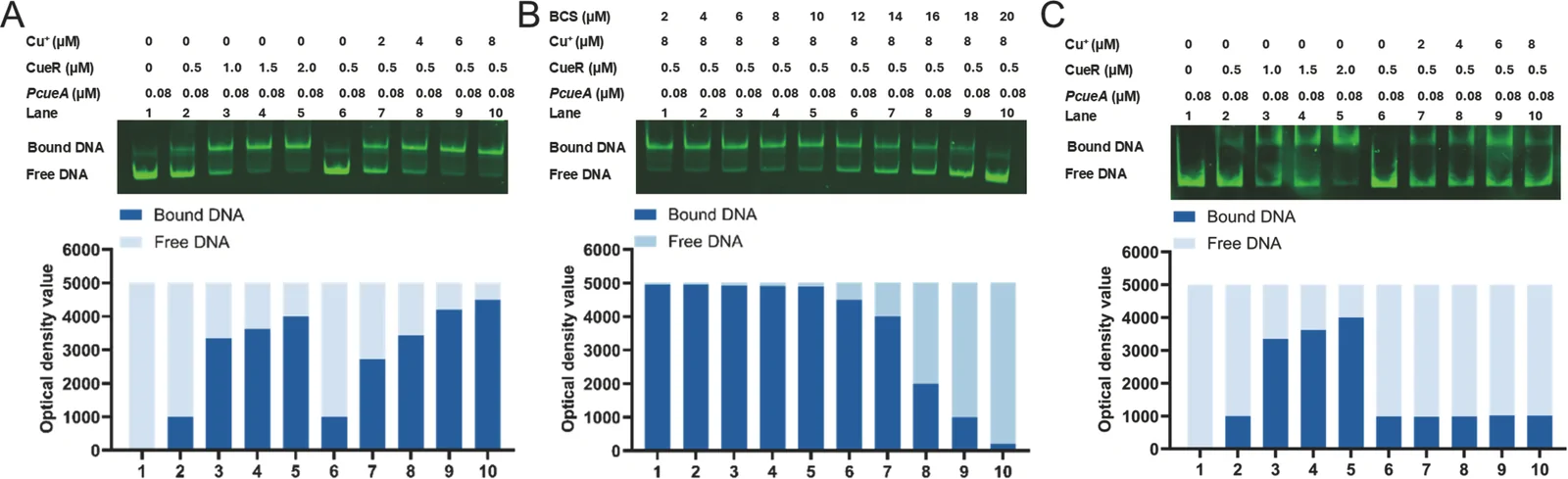
Cu^+^ enhances the binding of CueR to the *PcueA* region. EMSA of CueR binding to the *cueA* promoter fragment. Purified CueR was incubated with a 6-FAM-labeled 300-bp *cueA* promoter fragment at 30°C for 30 min. Complexes were resolved by electrophoresis on a 6% (wt/vol) non-denaturing polyacrylamide gel in 0.5 × TBE buffer at 4°C. (**A**) Lanes 1–5: binding reactions in the absence of Cu^+^. Lanes 6–10: binding reactions in the presence of added Cu^+^. To maintain a reducing environment, 10 mM ascorbic acid was included during sample incubation when Cu^+^ was added. (**B**) Lanes 1–10: binding reactions in the presence of 8 µM Cu^+^ together with 2–20 µM BCS. All lanes contained 0.3 µg of the *cueA* promoter fragment. Band intensities were quantified using Tana Image software (Tanan, China). Dark blue bars represent the optical density of the protein-bound DNA fragment; light blue bars represent the optical density of the free DNA fragment. (**C**) Add protein as CueR mutant protein, with other additives the same as panel (**A**).

For electrophoretic mobility shift assay (EMSA), a fluorescently labeled *PcueA* fragment (0.08 µM) was incubated with increasing amounts of CueR protein (0–2.0 µM). To assess the effect of Cu^+^, 0–8 µM Cu^+^ was added to the binding reactions prior to incubation, with 10 mM ascorbic acid present to maintain copper in its monovalent state. The results (Fig. 4A) show that CueR directly binds to the *PcueA* fragment, as evidenced by a decrease in free DNA intensity and a corresponding increase in bound DNA intensity with increasing amounts of CueR (lanes 1–5). Notably, the addition of 0–8 µM Cu^+^ enhanced the DNA-binding activity of CueR (lanes 6–10). To further confirm that this enhancement is Cu^+^-specific, the Cu^+^ chelator bathocuproinedisulfonic acid disodium salt (BCS) was included together with Cu^+^ in competitive binding assays. As shown (Fig. 4B), increasing BCS concentrations progressively restored the binding affinity of CueR to the level observed in the absence of Cu^+^. To further determine the effect of Cu^+^ on the binding affinity of CueR to the *PcueA* fragment, we substituted the cysteine residues at positions 112 and 121 of the Cu^+^-binding site in CueR with serines and repeated the EMSA assay. The results (Fig. 4C) showed that in the absence of Cu^+^, these mutations had no significant effect on the binding affinity between CueR and the *PcueA* fragment. Furthermore, while keeping the amounts of mutant CueR and *PcueA* fragment constant, the addition of varying concentrations of Cu^+^ did not alter the binding affinity of the mutant CueR to the *PcueA* fragment, which contrasts with the enhanced binding observed in Fig. 4A (lanes 6–10). Collectively, these results demonstrate that CueR regulates *cueA* expression through direct promoter binding, and that Cu^+^ enhances CueR-DNA complex formation.

### *cueR* and *cueA* form an operon *cueRA* to regulate copper ion transport

**Fig 5:**
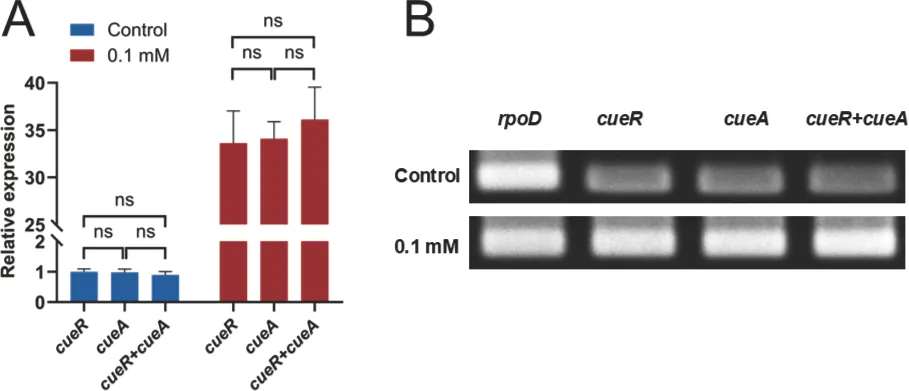
Consistent transcription levels of *cueR* and *cueA* across varying copper concentrations**.** (**A**) Relative expression levels of *cueR*, *cueA*, and *cueR + cueA* in the WT strain under 0 vs 0.1 mM Cu²^+^. (**B**) Agarose gel electrophoresis of qRT-PCR amplicons. Data are shown as mean ± SD (*n* = 3). Two-tailed unpaired Student’s *t*-test was used to calculate *P* values. ns, not significant.

Analysis of the ORFs of *cueR* and *cueA* revealed a 4-bp overlap between the two genes, with only one promoter region identified upstream of *cueR* and *cueA*. Thus, we hypothesized that *cueR* and *cueA* form an operon *cueRA*. To verify this, qRT-PCR primers were designed for the ORFs of *cueR* and *cueA*, and a pair of specific primers was designed to amplify a 300-bp fragment (*cueR + cueA*) spanning the 150 bp at the downstream of *cueA* and the 150 bp at the upstream of *cueR*. The expression levels of *cueR*, *cueA*, and *cueR + cueA* in WT strains treated with 0.1 mM CuSO₄·5H₂O were determined, with PBS-treated cells serving as the control. The data showed (Fig. 5A and B) that in the control group without Cu²^+^ stress, the transcriptional levels of *cueA*, *cueR*, and the overlapping *cueR + cueA* fragment were consistent with no significant differences. After treatment with 0.1 mM CuSO₄·5H₂O, the transcription of *cueA*, *cueR*, and the *cueR + cueA* was synchronously upregulated, yet their transcript levels remained consistent, showing no significant difference from each other. These results confirm that *cueA* and *cueR* are co-transcribed and form an operon *cueRA* that regulates copper transport in *P. chlororaphis* zm-1.

### Disruption of copper homeostasis impairs peanut root colonization by *P. chlororaphis* zm-1

To further investigate the relationship between copper homeostasis and the colonization capacity of *P. chlororaphis* zm-1, we conducted a greenhouse pot experiment to evaluate the colonization ability of Δ*cueR*, Δ*cueA*, Δ*cueRA*, Δ*cueR::cueR*, Δ*cueA::cueA*, and wild-type strains in peanut roots.

**Fig 6:**
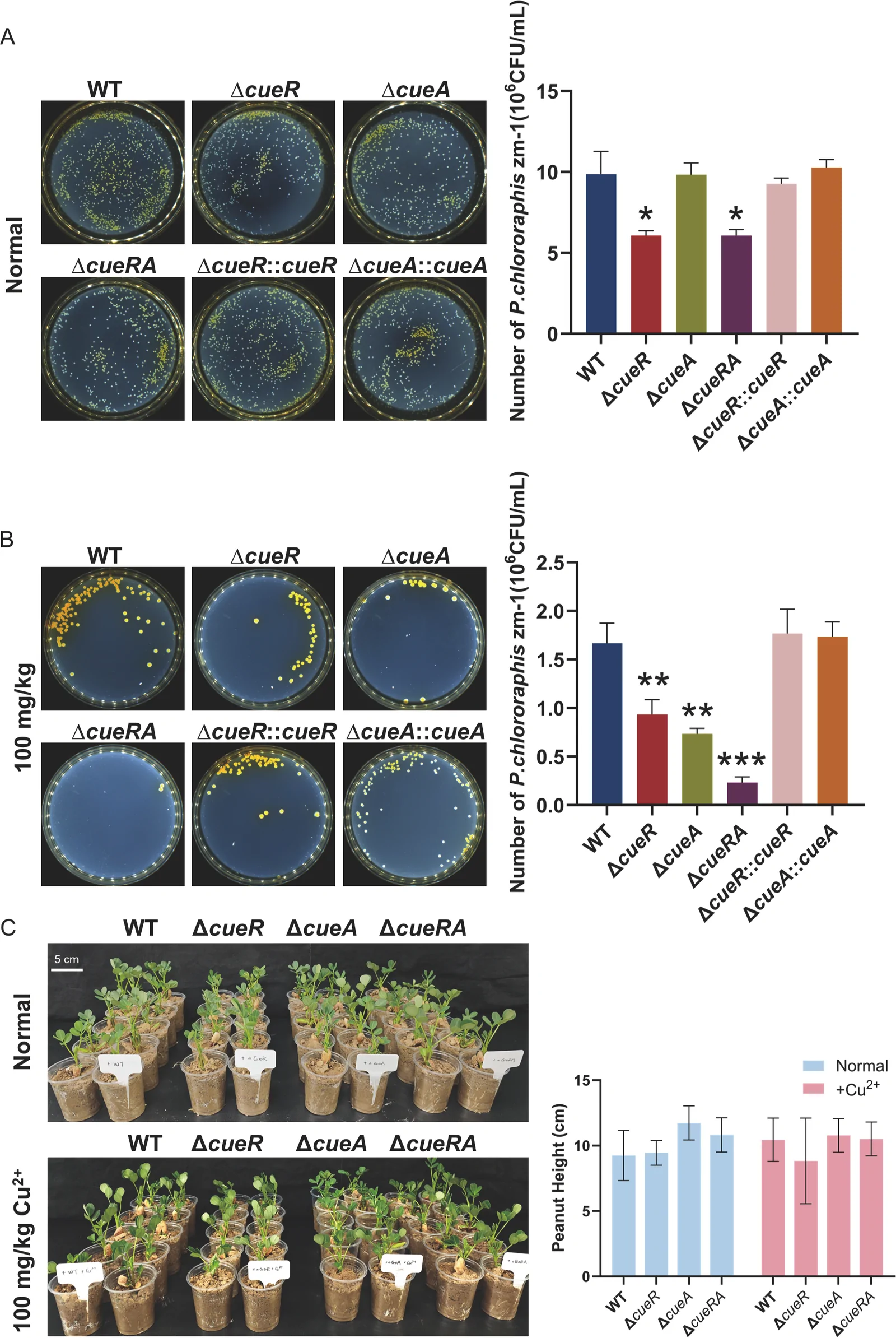
Copper homeostasis imbalance impairs the ability of *P. chlororaphis* zm-1 to colonize peanut roots. (**A**) Colonization of peanut roots by *P. chlororaphi*s zm-1 strains in untreated soil. (**B**) Colonization of peanut roots by *P. chlororaphis* zm-1 strains in copper-supplemented soil (100 mg/kg). Peanuts inoculated with *P. chlororaphis* zm-1 were grown for 14 days in soil with either native or excess copper levels (100 mg/kg). Roots were then harvested, washed with sterile water, weighed, and homogenized. The homogenate was resuspended in 5 mL of PBS, and 1 mL of the suspension was serially diluted to 10^-^³ and plated on LB agar supplemented with chloramphenicol (30 µg/mL). (**C**) Statistical analysis of peanut plant height growth. Measure the distance from the peanut cotyledon to the tip of the peanut. Data were represented as the mean ± SD (*n* = 3). Two-tailed unpaired Student’s *t*-test was used to calculate *P* values. *P < 0.5, **P < 0.1, ***P < 0.001.

Colonization results (Fig. 6A) showed that under normal soil conditions, the colonization capacity of Δ*cueA* (9.83 ± 0.72 × 10⁶ CFU/mL) was not significantly different from that of WT (9.86 ± 1.40 × 10⁶ CFU/mL). However, the Δ*cueR* and Δ*cueRA* strains reached colonization levels of only (6.06 ± 0.30) × 10⁶ CFU/mL and (6.06 ± 0.37) × 10⁶ CFU/mL, representing a 38% decrease compared to the WT. The colonization capacity of Δ*cueR::cueR* (9.26 ± 0.35 × 10⁶ CFU/mL) was restored to the WT level, suggesting that CueR may be involved in other physiological processes related to colonization in addition to copper homeostasis regulation.

To investigate colonization capacity under copper stress, soil was spiked with copper to a concentration of 100 mg/kg. The results showed (Fig. 6B) that under copper stress, the colonization capacities of Δ*cueR* (0.93 ± 0.15 × 10⁶ CFU/mL), Δ*cueA* (0.73 ± 0.05 × 10⁶ CFU/mL), and Δ*cueRA* (0.23 ± 0.05 × 10⁶ CFU/mL) were all significantly reduced in a gradient manner compared to WT (1.66 ± 0.20 × 10⁶ CFU/mL), with decreases of 44%, 56%, and 86%, respectively. The colonization capacities of Δ*cueR::cueR* (1.76 ± 0.25 × 10⁶ CFU/mL) and Δ*cueA::cueA* (1.73 ± 0.15 × 10⁶ CFU/mL) were restored to the WT level. These results indicate that the colonization capacities of the Δ*cueR*, Δ*cueA*, and Δ*cueRA* deletion mutants decrease in a gradient under copper stress, which is consistent with their growth trends under copper stress. There was no significant difference in the growth of peanuts in the colonization experiment (Fig. 6C).

## DISCUSSION

Copper, as an essential trace element and potential toxic factor for microorganisms, and its homeostasis regulatory mechanisms are core to maintaining bacterial survival and functional performance. Bacteria have evolved multiple copper homeostasis systems (21, 37), among which the copper efflux pathway mediated by the MerR family transcription factor CueR has emerged as a research hotspot in recent years due to its critical role in pathogenic bacterial drug resistance (23, 38). However, current studies mainly focus on human pathogens such as *P. aeruginosa* and *Vibrio cholerae* (39, 40), and the molecular characteristics, working modes of this system in plant biocontrol bacteria, as well as how the regulatory mechanisms adapt to the dynamic rhizospheric copper environment and biocontrol functional requirements, remain to be further elucidated. *P. chlororaphis* zm-1 is a highly efficient biocontrol strain isolated by our laboratory. Plate confrontation assays verified its strong inhibitory activity against diverse crop pathogenic fungi, e.g., *S. rolfsii*, *Rhizoctonia cerealis*, *Rhizoctonia solani*, and *Fusarium oxysporum fsp. niveum* (Fig. S5), while field trials showed that its control efficacy against peanut stem rot (57.09%) exceeded that of the conventional fungicide carbendazim (49.87%) (36). Notably, the strain’s colonization capacity in copper-contaminated farmlands directly dictates its control performance against soil-borne diseases (41). In this study, we systematically analyzed the genetic structure, regulatory mode, and physiological functions of the CueR-CueA module using *P. chlororaphis* zm-1 as the research object. This work not only provides new theoretical insights into the diversity of copper homeostasis regulation in *Pseudomonas* but also establishes a cross-boundary association of “heavy metal regulation—rhizospheric adaptation—biocontrol efficacy,” offering molecular targets and scientific evidence for the targeted improvement of biocontrol bacteria in contaminated soils.

Integrated analysis of bioinformatics predictions confirmed the core functional conservation of CueR and CueA in *P. chlororaphis* zm-1. Sequence analysis revealed that CueR from *P. chlororaphis* zm-1 shares 44% sequence identity with *E. coli* K-12 CueR and possesses a C-terminal copper-binding domain containing the conserved dual cysteine residues (Cys112 and Cys120) characteristic of MerR family regulators. As a P_1B_-type ATPase, CueA harbors the conserved CPC motif in its transmembrane region. This motif serves as a core functional site for copper ion transport (42, 43) and is identical to sequences found in known copper efflux pumps (11, 12). Notably, phylogenetic tree analysis (Fig. S1A) revealed that CueR from *P. chlororaphis* zm-1 forms an independent branch compared to rhizosphere-promoting *Pseudomonas* strains (e.g., *P. fluorescens* SBW25, *P. putida* PNL-MK25). This suggests that selection pressure from the rhizospheric habitat may drive the adaptive evolution of CueR (44, 45).

The co-transcribed operon structure of *cueRA* revealed by genetic structure analysis is a core molecular feature that distinguishes the copper regulatory system of *P. chlororaphis* zm-1 from that of pathogenic bacteria. Bioinformatics predictions indicated that there is only one promoter region upstream of *cueR* and *cueA*, with a 4-bp overlap between their ORFs; combined with qRT-PCR results (Fig. 5A and B), this directly confirms that they form a co-transcriptional unit. This unique co-transcription unit has only been similarly reported in *Pseudomonas putida*, which markedly differs from the independent transcription pattern in *E. coli* (46). In *E. coli, cueR* exists as a monocistron, whereas *copA* is separated by the *ybaST* operon (47). The ecological advantage of this co-transcriptional structure can be explained by the characteristics of the rhizospheric environment: the copper concentration in rhizosphere soil fluctuates dramatically due to root exudates, microbial metabolism, and other factors (48). The coordinated expression of regulatory factors and effector genes avoids the regulatory delay between “signal perception and effector execution”—upon sensing copper signals, CueR can synchronously initiate the synthesis of itself and CueA, enabling rapid assembly and function of the copper efflux pump. This ensures the effective colonization of bacteria in heavy metal environments (49). Further promoter sequence analysis showed that the palindromic sequence bound by CueR (5′-ACCTTGCCATGCTGGCAAGGT-3′) differs by 7 bp from the recognition sequence in *E. coli* (5′-ACCTTCCCCTTGCTGGAAGGT-3′). Additionally, existing studies have confirmed that the binding specificity of MerR family proteins is jointly determined by the base composition of the palindromic sequence and the conformation of the spacer region (50), suggesting that this difference may alter the binding affinity of CueR. This structural specialization may serve as the molecular basis for *P. chlororaphis* zm-1 to accurately respond to copper signals in complex rhizospheric environments and avoid cross-reactions with other metal regulatory systems.

Integrating qRT-PCR and EMSA, our study reveals that CueR drives *cueA* expression through a unique copper-dependent positive regulatory mechanism. Copper stress gradient experiments showed that when the Cu²^+^ concentration increased from 0.1 mM to 0.2 mM, the expression level of *cueA* in the WT strain increased exponentially (upregulated by 89-fold), while the *cueA* expression level in the Δ*cueR* strain remained at a basal level (only 0.3-fold of that in the WT under no copper stress) (Fig. 3A). The complementation strain fully restored the regulatory ability, confirming that CueR is an essential factor for the transcriptional activation of *cueA*. EMSA experiments further demonstrated that CueR can bind to the promoter in the absence of copper, and its binding ability is significantly enhanced upon copper ion addition; however (Fig. 4A), no such enhancement was observed for the mutant CueR with disrupted Cu^+^-binding sites when Cu^+^ was added (Fig. 4C). This is different from the regulation mode in *E. coli*, where “CueR binds to the promoter region in the absence of copper, but its binding ability decreases when copper is bound” (51). From the perspective of ecological adaptability, this positive regulation mode has significant advantages: when copper in the rhizosphere environment is below the CueR-binding threshold, the unactivated CueR enables *cueA* to maintain low-level expression; upon exposure to copper stress, the binding of Cu^+^ to CueR enhances its DNA-binding affinity, enabling the high-level expression of *cueA*. This dual-mode regulation, which coordinates basal defense and emergency activation, is key to how rhizosphere microbes adapt to fluctuating environments.

Copper tolerance experiments and intracellular copper quantification analysis constructed a functional chain of “CueR regulation—CueA efflux—copper homeostasis maintenance,” providing direct physiological evidence for the regulatory mechanism. Growth curve determination showed that the WT strain could still maintain a certain level of growth (OD₆₀₀ reaching 0.4) at 0.3 mM Cu²^+^, while the Δ*cueRA* double deletion mutant exhibited significant growth inhibition at 0.1 mM Cu²^+^ (Fig. 2A). Spot dilution assays further confirmed the gradient difference in bacterial copper tolerance: WT > Δ*cueR* > Δ*cueA* > Δ*cueRA* (Fig. 2B). Atomic absorption spectrometry quantification results showed that this growth difference results from the intracellular accumulation of copper: the intracellular copper content of the Δ*cueRA* strain (1.49 mg/g dry cell) was 3.1-fold higher than that of the WT (0.48 mg/g dry cell), the Δ*cueA* strain (1.30 mg/g dry cell) was slightly lower than the Δ*cueRA* mutant, and the Δ*cueR* strain (0.89 mg/g dry cell) was significantly higher than the WT (Fig. 2C). This result can be explained by the synergistic effect of the regulatory mechanism and gene functions: the Δ*cueA* strain lacks the CueA efflux pump, leading to intracellular copper accumulation and the weakest tolerance; the Δ*cueR* strain can express CueA at a basal level (qRT-PCR confirmed no significant difference in *cueA* expression between Δ*cueR* and WT in the absence of copper) but cannot activate *cueA* upregulation under copper stress, resulting in insufficient efflux efficiency and moderate intracellular copper accumulation; the WT and complementation strains can achieve high-efficiency expression of CueA through the positive regulation of CueR, rapidly efflux excess copper, and maintain intracellular homeostasis.

**Fig 7:**
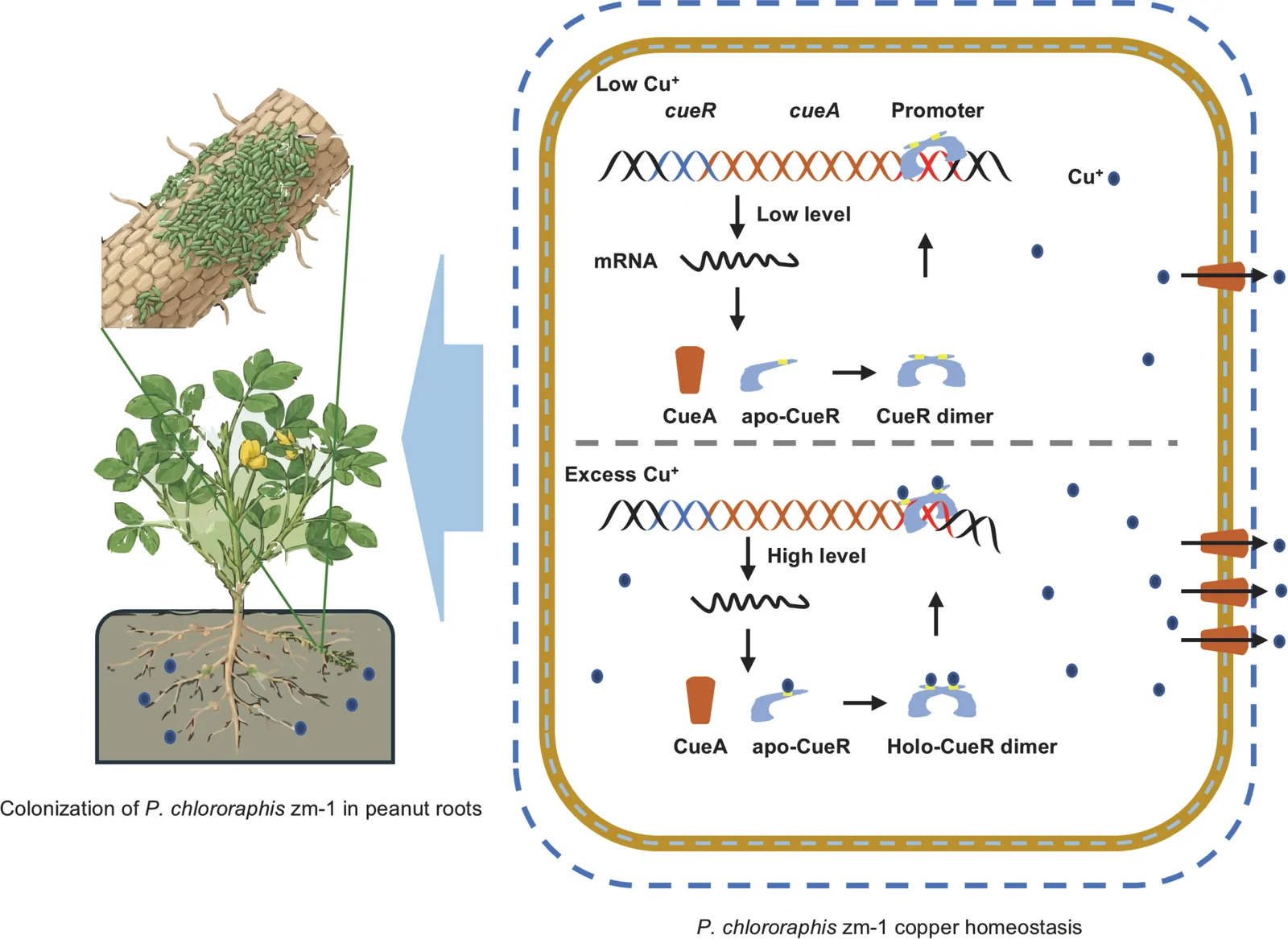
Model of the *cueRA* operon-mediated copper homeostasis mechanism in *P. chlororaphis* zm-1. In *P. chlororaphis* zm-1, *cueR* and *cueA* form the *cueRA* operon. under low-copper conditions, a small amount of CueR dimer binds to the promoter region, resulting in a low level of transcription. In the presence of excess copper, the CueR dimer bound to Cu^+^ drives high-level transcription of the *cueRA* operon, producing a large amount of CueA to export copper ions. The maintenance of copper homeostasis ensures that *P. chlororaphis* zm-1 can stably colonize peanut roots when environmental copper levels are excessively high. The red segment of the DNA double helix represents the promoter region of the *cueRA* operon, the brown segment corresponds to the *cueA* open reading frame (ORF), and the blue segment corresponds to the *cueR* ORF. The translated proteins are colored to match their respective ORFs. The wavy line denotes the mRNA transcript. The yellow region on the CueR protein indicates the Cu^+^ binding site, and the light-blue sphere represents Cu^+^.

Based on the above phenotypic determinations and molecular biological experimental evidence, we also propose a basic model for the regulation of copper ion efflux by the operon composed of *cueR* and *cueA* in *P. chlororaphis* (Fig. 7). Specifically, when the environmental copper ion concentration is below the threshold, Cu-free CueR binds to the promoter region as a dimer. When the environmental copper ion concentration exceeds the threshold, copper binding enhances the affinity of CueR for the promoter region, thereby driving the rapid transcription of the *cueRA* operon. This maintenance of copper homeostasis subsequently allows *P. chlororaphis* zm-1 to better colonize the peanut rhizosphere.

In addition to systematically analyzing the molecular mechanism by which the CueR-CueA system regulates copper ion efflux in *P. chlororaphis* zm-1, this study also verified that this system modulates the rhizosphere colonization ability of biocontrol bacteria, establishing a cross-boundary association between “heavy metal regulation and biocontrol function.” Hierarchical analysis of peanut root colonization experiments revealed a dual regulatory mechanism: under normal soil conditions, the colonization capacity was significantly impaired (38% reduction) in both Δ*cueR* and Δ*cueRA* mutants under normal soil conditions (Fig. 6A), highlighting a specific requirement for CueR, not CueA, in this process. This excludes the impact of copper homeostasis disruption, suggesting that CueR has colonization-regulating functions beyond copper regulation; under copper-contaminated soil (100 mg/kg), the colonization abilities of deletion strains were significantly reduced, with the magnitude of reduction consistent with their copper tolerance (Δ*cueRA*: 86% reduction > Δ*cueA*: 56% reduction > Δ*cueR*: 44% reduction) (Fig. 6B), confirming that copper homeostasis is a core guarantee for colonization in contaminated environments. Consistent with that “rhizosphere colonization is a prerequisite for biocontrol bacteria to exert their effects,” this result highlights the application value of the CueR system—strengthening this system can simultaneously improve the heavy metal tolerance and rhizosphere colonization efficiency of biocontrol bacteria. Furthermore, transcriptomic screening of *P. fluorescens* SBW25 under plant-associated conditions confirmed the expression of both *cueR* and *cueA* (52). A separate study demonstrated the involvement of *cueA* in the rhizosphere colonization process of strain SBW25 (44). These findings suggest that CueR-mediated regulation of colonization may represent a conserved trait within the *Pseudomonas* genus, while the present study provides new mechanistic insights into this cross-strain phenomenon.

Although this study has explored the core functions of the *cueRA* operon, there are still scientific issues that require in-depth investigation, which also point out directions for future research. First, the regulatory network of CueR needs to be expanded. In *Pseudomonas aeruginosa* PAO1, CueR possesses five binding sites located upstream of *copA*1, *copZ*1, *copZ*2, *cusCAB*, and the PA3515–3519 cluster encoding glycolysis and fatty acid metabolism (53, 54). We employed BLAST searches to identify homologous proteins within the *P. chlororaphis* zm-1 proteome. Consequently, a protein designated as WP_007923288.1 was selected due to its 78.5% amino acid sequence identity with a cytosolic copper chaperone in *P. fluorescens*; this protein was tentatively named CueZ. Although the constructed Δ*cueZ* mutant exhibited no significant growth difference compared to the wild-type strain under copper stress, it showed slightly compromised growth specifically under 0.3 mM Cu^2+^ stress (Fig. S6A). Notably, intracellular copper quantification revealed that the internal copper level of the Δ*cueZ* strain decreased from 0.390 ± 0.027 in the wild-type to 0.269 ± 0.03 (Fig. S6B). These findings indicate that CueZ functions in copper storage within *P. chlororaphis* zm-1. This aligns with our BLAST results, where CueZ shared a high sequence identity of 66.1% with CopZ2, a known copper pool protein in *P. aeruginosa*. However, the search results for other candidates were unsatisfactory; analysis of their promoter regions revealed a lack of the palindromic sequences required for CueR binding. Second, the molecular mechanism of CueR’s dual functions requires further elucidation. Identifying direct target genes through relevant experiments to distinguish its “copper regulatory targets” and “colonization regulatory targets” will clarify the molecular basis of its multiple functions. Third, field-scale functional verification needs to be supplemented. The colonization experiments in this study were conducted at the greenhouse pot level; future studies should verify the colonization efficiency and disease control effect of engineered strains in actual copper-contaminated farmlands to evaluate their application potential.

In summary, this study systematically revealed the “working structure—positive regulation—dual functions” characteristics of the CueR-CueA module in biocontrol bacteria *P. chlororaphis* zm-1. We have verified the existence of the co-transcribed *cueRA* operon in *P. chlororaphis*, elucidate the positive regulatory mechanism by which CueR enhances promoter binding under copper signals, establish a complete functional chain of “genetic structure—regulatory mode—copper tolerance—rhizosphere colonization,” and discover the novel colonization-regulating function of CueR. This study enriches the understanding of the diversity of bacterial copper homeostasis regulation, reveals the rhizosphere habitat-driven differentiation of regulatory mechanisms, establishes a bridge between heavy metal regulatory systems and biocontrol functions, and provides a new paradigm for analyzing the environmental adaptability mechanisms of biocontrol bacteria. Meanwhile, it clarifies the core value of CueR as a target for biocontrol bacteria improvement, offering molecular basis for the targeted breeding of biocontrol bacteria in contaminated soils. Future research will focus on the elucidation of the CueR regulatory network and field verification of engineered strains, further promoting the application of biocontrol bacteria in the bioremediation of contaminated soils.

## MATERIALS AND METHODS

### Strains and culture conditions

The WT strain *P. chlororaphis* zm-1 used in this study was isolated from the rhizosphere of *Anemarrhena asphodeloides* grown in the experimental field of the College of Life Sciences, Henan University. The WT strain has been identified by 16S rRNA sequencing and fully sequenced for its whole genome (GeneBank ID: CP048051.1). Bacterial strains were grown in Lysogeny Broth (LB: 10 g/L tryptone, 5 g/L yeast extract, 10 g/L NaCl) at 30°C for growth assays and related experiments. *E. coli* were cultured in LB at 37°C. For solid medium, LB was supplemented with 1.5% (wt/vol) agar. Required antibiotics were added: 100 µg/mL ampicillin (Amp), 30 µg/mL chloramphenicol (Cm), and 50 µg/mL kanamycin (Kan). All antibiotics and other biochemical reagents, including isopropyl-β-D-thiogalactopyranoside (IPTG), were purchased from Sangon Biotech (Shanghai, China). In this study, *E. coli* 116 (*pir*^+^) was used for plasmid cloning, *E. coli* S17-1 (*λpir*) for bacterial conjugation, and *E. coli* BL21 (DE3) for CueR protein expression. Detailed information of the strains and plasmids used in this experiment is listed in Table S1.

### Bioinformatics analysis

Nucleotide and protein sequences required in this study were downloaded and analyzed from the NCBI database (http://www.ncbi.nlm.nih.gov). Protein domain analysis was performed using the online tool SMART (http://smart.embl-heidelberg.de/). Amino acid sequence alignment among different species and phylogenetic tree construction were conducted using MEGA 7.0 (55). The amino acid sequence alignment results were further optimized using the online tool ESPript 3.0 (https://espript.ibcp.fr/ESPript/ESPript/index.php). Protein three-dimensional structure prediction was carried out via the online tool AlphaFold server (https://alphafoldserver.com/), and the predicted results were sorted and visualized using PyMOL (https://pymol.org/2/support.html). The molecular weight of the protein was predicted using the online software ExPASy (https://web.expasy.org/compute_pi/). Gene promoter region prediction was analyzed using BPROM (https://www.softberry.com/berry.phtml).

### Construction of gene deletion and complementation strains

Gene deletion strains of *P. chlororaphis* zm-1 were constructed by the previously described homologous recombination exchange method (56). Specifically, the upstream (900 bp) and downstream (900 bp) regions of the target gene were amplified by PCR using the genome of *P. chlororaphis* zm-1 as the template, and the deletion primers are listed in Table S2. Taking the deletion of *cueR* as an example, 900 bp upstream (using primers *cueR*-up-*Eco*RI-s*/cueR*-up-*Bam*HI-a) and downstream (using primers *cueR*-down-*Bam*HI-s/*cueR*-down-*Hin*dIII-a) fragments of the *cueR* were amplified from the genome of *P. chlororaphis* zm-1. The sequences were digested with restriction endonucleases *Eco*RI-*Bam*HI and *Bam*HI-*Hin*dIII and then ligated into the *Eco*RI-*Hin*dIII site of plasmid pK18mob *sacB* (5,719 bp) (Fig. S2A) using T4 DNA ligase to generate the 7.5 kb recombinant plasmid *cueR*-pK18mob *sacB*. This plasmid was transformed into *E. coli* 116 (*pir*^+^) by heat shock for cloning and then electroporated into *E. coli* S17-1 (*λpir*). Finally, the recombinant plasmid *cueR*-pK18mob *sacB* was transferred into *P. chlororaphis* zm-1 via bacterial conjugation with *E. coli* S17-1 (*λpir*) for homologous recombination (Fig. S2B). Clones with a chromosomal insertion of the *cueR*-pK18mob *sacB* were selected on LB agar plates supplemented with 30 µg/mL Cm and 50 µg/mL Kan. Strains with homologous recombination were screened on LB plates supplemented with 30 µg/mL Cm and 15% sucrose, and the *cueR* deletion mutant was verified using primers *cueR*-up-*Eco*RI-s and *cueR*-down-*Hin*dIII-a (Fig. S2C). The *cueA*, *cueRA,* and *cueZ* deletion mutants were constructed using the same method. All plasmids and gene deletion strains were confirmed by DNA sequencing (Sangon Biotech, Shanghai, China).

For the construction of complementation strains, the DNA sequence containing the promoter and ORF of the target gene was amplified with specific primers and ligated into the vector pK18mob *sacB*YS (Fig. S3A). The recombinant vector was then introduced into the corresponding gene deletion mutant via homologous recombination to obtain the complementation strain (Fig. S3B), following the same procedure as gene deletion. All gene deletion and complementation strains were further verified by qRT-PCR (Fig. S2D and S3D).

### RNA extraction and qRT-PCR

Overnight-cultured test strains were inoculated into fresh LB medium at a 1:100 dilution, supplemented with 30 µg/mL Cm, and cultured at 30°C with shaking at 220 rpm for 6 h to reach mid-exponential phase (OD₆₀₀ = 0.5). The culture was then challenged with filter-sterilized stock solution of CuSO₄·5H₂O and incubated for an additional 30 min. Two milliliters of the culture was collected by centrifugation, and total RNA was extracted using a total RNA extraction kit (Vazyme, China). The quantity and quality of total RNA were evaluated by measuring OD₂₆₀ and OD₂₈₀ values using a Nanodrop spectrophotometer (Thermo Fisher Scientific, USA). cDNAs were synthesized using the Fast Quant RT Kit (Tiangen, China) according to the manufacturer’s protocol. Quantitative reverse transcription polymerase chain reaction was performed using GoTaq qPCR master mix (Promega, USA) on an ABI 7500 Fast Cycler (Thermo Fisher Scientific, USA) following the manufacturer’s recommendations. The RNA polymerase sigma factor *rpoD* was used as the internal reference gene, and data were analyzed using the 2^−∆∆*Ct*^ method (57). For each sample, three independent biological replicates were performed.

### Expression and purification of CueR protein

The full-length 402 bp fragment with restriction enzyme sites was amplified from the *P. chlororaphis* zm-1 genome using primers *cueR—Bam*HI-s and *cueR—Xho*I-a, then ligated into the pGEX-6p-1 expression vector to construct the CueR protein expression vector. The recombinant vector was electroporated into *E. coli* BL21 (DE3). Bacteria were cultured in LB supplemented with 100 µg/mL Amp to exponential phase (OD₆₀₀ = 0.6), induced with 0.1 mM IPTG, and further cultured at 16°C for 16 h. Harvested bacteria were resuspended in buffer (50 mM Tris-HCl [pH 7.5], 150 mM NaCl, and 10 mM PMSF), lysed by ultrasonic disruption, and centrifuged at 10,000 rpm for 1 h. The supernatant was filtered through a 0.45 μm pore size filter and loaded onto a GST-tag affinity gravity column at a flow rate of 2 mL/min. The column was washed with 10 column volumes of PBS buffer, and the GST-tagged CueR protein was eluted with Elution Buffer (50 mM Tris-HCl [pH 7.5], 10 mM GSH). The purified sample was stored at −80°C in buffer containing 50 mM Tris-HCl (pH 7.0), 150 mM NaCl, and 30% glycerol (58).

To obtain CueR protein without the GST tag, the GST-tagged CueR protein sample was dialyzed against digestion buffer (50 mM Tris-HCl [pH 7.5], 150 mM NaCl, 1 mM EDTA, 1 mM DTT) for 12 h. PreScission protease (Beyotime, China) was added at a ratio of 20 U/mg protein, and the mixture was incubated at 16°C for 20 h to remove the GST tag. The digested reaction mixture was passed through a GST-tag affinity gravity column to remove the GST tag and undigested protein, and the flow-through containing the CueR protein was collected. The protein concentration was determined using a BCA protein assay kit (Thermo Fisher Scientific, USA).

To obtain the CueR mutant protein, we introduced mutations into the primer design to substitute guanine (G) with cytosine (C) at positions 335 and 359 of the *cueR* ORF via PCR, which resulted in the substitution of cysteine with serine at positions 112 and 121 of the mutated CueR protein. Subsequent experimental procedures for the CueR mutant protein remained identical to those used for the wild-type CueR protein.

### Electrophoretic mobility shift assay

DNA probes used for EMSA were prepared by PCR from the pMD-19 vector carrying the *PcueA* region. Specifically, a 300 bp fragment containing the *PcueA* region (spanning from + 50 bp downstream to −250 bp upstream of the start codon) was amplified from the *P. chlororaphis* zm-1 genome by PCR and then ligated into the pMD-19 vector. Fluorescently labeled DNA probes were generated by PCR using 6-FAM-labeled specific primers synthesized by Sangon Biotech (Shanghai, China). The concentration of DNA was determined using a Nanodrop spectrophotometer (Thermo Fisher Scientific, USA). Different amounts of CueR protein were mixed with 10 μL of reaction mixture containing 0.08 μm labeled probe, 20 mM Tris-HCl (pH 8.0), 50 μg/mL BSA, 50 mM NaCl, 1 mM DTT, 5 μg/mL salmon sperm DNA, and 5% glycerol, with a final volume of 20 μL. To determine the effect of Cu^+^ on the promoter-binding ability of CueR, 10 mM ascorbic acid was added to maintain a reducing environment, and BCS was used to chelate Cu^+^. Samples were incubated at 30°C for 30 min and then electrophoresed on a 6% native polyacrylamide gel in 0.5 × Tris-borate-EDTA buffer (TBE, 45 mM Tris-boric acid, 1 mM EDTA) at 100 V and 4℃ for 2 h. The gel was scanned using a gel imager (Tanan, China), and the intensity of gel bands was quantified using Tanan Image software (Tanan, China). The EMSA workflow for the CueR mutant protein remained identical to that used for the wild-type CueR protein.

### Plate stress assay

A single colony was picked and inoculated into 5 mL LB medium and cultured overnight at 30°C with shaking at 220 rpm. One milliliter of the overnight culture was collected by centrifugation, and the bacterial cells were washed with PBS and resuspended in different volumes of PBS to an OD₆₀₀ of 0.6. Serial dilutions of the bacterial suspension were inoculated onto LB agar plates supplemented with different concentrations of CuSO₄·5H₂O and then incubated at 30°C for 24 h for observation. Thirty micrograms per milliliter of Cm was added to the LB agar to prevent contamination by miscellaneous bacteria.

### Growth curve determination

Adjust overnight culture from test tube to an OD₆₀₀ of 0.6 with PBS. The bacterial suspension was inoculated into LB medium at a 1:100 dilution, supplemented with different concentrations of CuSO₄·5H₂O and 30 µg/mL Cm. Growth curves were determined using a Bioscreen growth curve analyzer (Bioscreen, Finland) at 30°C with shaking at 220 rpm, and the OD₆₀₀ value was measured every 2 h for 24 h. Each experimental group was set with three biological replicates.

### Determination of intracellular copper ion levels

Overnight-cultured bacteria in test tubes were inoculated into fresh LB medium at a 1% inoculum, supplemented with 30 µg/mL Cm, and cultured with shaking until logarithmic phase. The culture was then challenged with 0.2 mM CuSO₄·5H₂O, and bacterial cells were collected by centrifugation at 6,000 rpm for 10 min after 2 h. The cells were first washed twice with PBS, treated with excess DTT and BCS (twice the concentration of CuSO₄·5H₂O), and then washed three times with Milli-Q water. Bacterial cells were oven-dried at 80°C to constant weight, dissolved in fuming nitric acid (trace metal grade), and transferred to digestion tubes for digestion at 120°C for 2 h. The digested solution was diluted appropriately, and the copper concentration was determined using an atomic absorption spectrometer (Agilent, USA).

### Peanut root colonization assay

Greenhouse pot experiments were conducted to evaluate the peanut root colonization ability of various deletion mutants of *P. chlororaphis* zm-1, following the method commonly used in the laboratory (36, 59). Briefly, peanut seeds were removed from their shells and then disinfected by soaking in 1% (vol/vol) sodium hypochlorite for 10 min, 1% (wt/wt) copper sulfate solution for 1 min, and 75% (vol/vol) ethanol for 30 s. Disinfected seeds were washed three times with sterile water and germinated in a sterile container at 30°C in the dark for 36 h. Strains to be tested were cultured overnight at 30°C with shaking at 220 rpm, collected by centrifugation at 6,000 × *g* for 3 min, washed twice with PBS, and resuspended in PBS to an OD₆₀₀ of 0.6. Seeds with consistent root lengths were soaked in the bacterial suspension at room temperature for 2 h and then sown into disposable plastic pots. The culture substrate was a mixture of local sand, peat soil, and vermiculite (3:2:1, vol/vol/vol), sterilized at 121°C for 30 min. Each experimental group included 5 samples with three replicates. Peanut plants were grown in an artificial climate chamber with a 14 h light/10 h dark cycle, day temperature of 28°C, night temperature of 25°C, and relative humidity of 70%. For soil copper stress treatment, copper sulfate was dissolved in water and mixed into the soil to a final copper concentration of 100 mg/kg. The copper stress level (100 mg/kg soil) was set with reference to the risk screening value for copper in agricultural soils as stipulated in the Chinese national standard GB 15618-2018 (for pH > 6.5) (48). After 14 days of cultivation, peanut plants were harvested, and roots were weighed, ground, serially diluted, and spread onto LB agar plates supplemented with 30 µg/mL Cm. Plates were incubated at 30°C for 2 days, and colony counting was performed using an automatic colony counter (Interscience, France) with manual calibration for accuracy.

### Statistical analysis

All experiments were performed in triplicate. Data were analyzed using GraphPad Prism 10.0 software (GraphPad Software, USA). Statistical significance was determined by Student’s *t*-test or by one-way ANOVA followed by Tukey-Kramer multiple comparisons test, where appropriate. Data are presented as means ± standard deviation (SD), and *P* < 0.05 was considered statistically significant.

## Supplementary Material

Reviewer comments

## Data Availability

The complete genome of *P. chlororaphis* zm-1 is available in GenBank (GenBank accession CP048051.1). All supporting data are provided in the Supplemental material.

## SUPPLEMENTAL MATERIAL

Supplemental figures and tables spectrum.03981-25-s0001.pdf Fig. S1 to S6; Tables S1 and S2.

OPEN PEER REVIEW reviewer-comments.pdf An accounting of the reviewer comments and feedback.

ASM does not own the copyrights to Supplemental Material that may be linked to, or accessed through, an article. The authors have granted ASM a non-exclusive, world-wide license to publish the Supplemental Material files. Please contact the corresponding author directly for reuse.
