# Supplementary material for: The transcriptional regulator CueR positively regulates the expression of the cueRA operon to maintain copper homeostasis in Pseudomonas chlororaphis zm-1
Source: Microbiol Spectr. 2026 Sep 17;14(10):e03981-25. doi: 10.1128/spectrum.03981-25 (PMC13688801; doi:10.1128/spectrum.03981-25)
Supplement: Supplementary file 1 [file reviewer-comments.pdf]

ASM | Microbiology Spectrum

The transcriptional regulator CueR positively regulates the expression of the *cueRA* operon to maintain copper homeostasis in *Pseudomonas chlororaphis* zm-1

Zhongkang Wang, Chen Li, Zhenming Lei, Sijia Duan, Feng Liu, Shaowei Wang, and Gang Wang

Corresponding Author(s): Gang Wang, Henan University College of Life Science

Review Timeline:

Submission Date:	December 9, 2025
Editorial Decision:	May 22, 2026
Revision Received:	July 17, 2026
Accepted:	August 19, 2026

Editor: Katharina Kujala

Reviewer(s): Disclosure of reviewer identity is with reference to reviewer comments included in decision letter(s). The following individuals involved in review of your submission have agreed to reveal their identity: Koch Hans-Georg (Reviewer #2)

Transaction Report:

(Note: With the exception of the correction of typographical or spelling errors that could be a source of ambiguity, letters and reports are not edited. The original formatting of letters and referee reports may not be reflected in this compilation.)

DOI: https://doi.org/10.1128/spectrum.03981-25

Re: Spectrum03981-25 (The transcriptional regulator CueR positively regulates the expression of the *cueRA* operon to maintain copper homeostasis in *Pseudomonas chlororaphis* zm-1)

Dear Dr. Gang Wang:

Thank you for the privilege of reviewing your work. Below you will find my comments, instructions from the Spectrum editorial office, and the reviewer comments.

Please return the manuscript within 60 days; if you cannot complete the modification within this time period, please contact me. If you do not wish to modify the manuscript and prefer to submit it to another journal, notify me immediately so that the manuscript may be formally withdrawn from consideration by Spectrum.

Revision Guidelines
To submit your modified manuscript, log into the submission site at https://spectrum.msubmit.net/cgi-bin/main.plex. Go to Author Tasks and click the appropriate manuscript title to begin. The information you entered when you first submitted the paper will be displayed; update this as necessary. Note the following requirements:

• Upload point-by-point responses to the issues raised by the reviewers in a file named "Response to Reviewers," NOT in your cover letter.
• Upload a compare copy of the manuscript (without figures) as a "Marked-Up Manuscript" file.
• Upload a clean .DOC/.DOCX version of the revised manuscript and remove the previous version.
• Each figure must be uploaded as a separate, editable, high-resolution file (TIFF or EPS preferred), and any multipanel figures must be assembled into one file.
• Any supplemental material intended for posting by ASM should be uploaded with their legends separate from the main manuscript. You can combine all supplemental material into one file (preferred) or split it into a maximum of 10 files with all associated legends included.

For complete guidelines on revision requirements, see our Submission and Review Process webpage. Submission of a paper that does not conform to guidelines may delay acceptance of your manuscript.

Data availability: ASM policy requires that data be available to the public upon online posting of the article, so please verify all links to sequence records, if present, and make sure that each number retrieves the full record of the data. If a new accession number is not linked or a link is broken, provide Spectrum production staff with the correct URL for the record. If the accession numbers for new data are not publicly accessible before the expected online posting of the article, publication may be delayed; please contact production staff (Spectrum@asmusa.org) immediately with the expected release date.

Publication Fees: For information on publication fees and which article types are subject to charges, visit our website. If your manuscript is accepted for publication and any fees apply, you will be contacted separately about payment during the production process; please follow the instructions in that e-mail. Arrangements for payment must be made before your article is published.

ASM Membership: Corresponding authors may join or renew ASM membership to obtain discounts on publication fees. Need to upgrade your membership level? Please contact Customer Service at Service@asmusa.org.

The ASM Journals program strives for constant improvement in our submission and publication process. Please tell us how we can improve your experience by taking this quick Author Survey.

Thank you for submitting your paper to Spectrum.

Sincerely,
Katharina Kujala
Editor
Microbiology Spectrum

Reviewer #1 (Comments for the Author):

This manuscript investigates the regulatory mechanism of CueR on copper homeostasis in Pseudomonas chlororaphis zm-1, a plant biocontrol bacterium. The authors identify that cueR and cueA form a cueRA operon, which is positively regulated by CueR in the presence of copper. This work provides insights into the diversity of copper homeostasis regulation in beneficial plant-associated bacteria. However, I have several concerns about the work.

1.The greenhouse pot experiments show that copper homeostasis imbalance impairs colonization in peanut rhizospheres. However, the current data mainly focuses on population counts. Did the authors observe any phenotypic changes in the peanut plants themselves when inoculated with the ΔcueR or ΔcueA mutants under copper stress compared to the wild type?
2.The study utilizes EMSA to verify that Cu^{+} enhances the binding of CueR to the cueA promoter region. While this demonstrates direct regulation, the binding affinity (Kd) is not explicitly calculated or discussed. Have the authors performed quantitative analyses, such as ITC or MST, to determine the binding affinity of CueR to the promoter in the presence versus the absence of Cu^{+}?
3.In Figure 2A, the labeling is insufficiently clear; for instance, it is difficult to distinguish which strain is represented by the orange line. Furthermore, the growth curves for the different strains are heavily intertwined, making them hard to read. For example, in the presence of 0.3 mM Cu^{2+}, the growth curve for the wild-type (WT) strain is completely obscured. I suggest improving the visual presentation, perhaps by separating the panels or using distinct markers to ensure all data points are visible.
4. I recommend redrawing the proposed model in Figure 7. The current illustration fails to clearly depict the mechanism by which Cu^{+} enhances the binding affinity of CueR to the cueA promoter, as demonstrated in your EMSA results. Additionally, the figure should explicitly illustrate the biological significance of CueR regulating CueA to provide a more comprehensive summary of your findings. Specifically, how this interaction facilitates copper efflux and maintains homeostasis.
5. Line 85, "soft" should be "soft".

Reviewer #2 (Comments for the Author):

Wang et al report on the role of the transcriptional regulator CueR in regulating copper homeostasis in Pseudomonas chlororaphis zm-1.
CueR belongs to the MerR family of transcriptional regulators and has been characterized in detail in Escherichia coli and Pseudomonas aeruginosa, whereas comparatively less is known about CueR in plant growth-promoting bacteria. Here, the authors identify the cueAR operon in Pseudomonas chlororaphis zm-1 and characterize its role in copper homeostasis. A similar operon organization has previously been reported in Pseudomonas putida (Adaikkalam & Swarup, 2002), and this study should therefore be cited in the relevant section. In light of these earlier findings, the novelty claims of the present manuscript should also be toned down accordingly.

Overall, this is a solid study that demonstrates that CueR in Pseudomonas chlororaphis zm-1 is an important regulator of copper homeostasis, primarily via regulating the expression of the CopA-like copper-exporting ATPase CueA. In contrast, the predicted role of CueAR in rhizosphere colonization is more speculative.

For improving the quality of the manuscript, the following issues should be addressed:

1. The more than 80-fold increase of cueA expression is not visible in Fig. 3B, this needs to be explained.

This also applies to Fig. 5B, where the about 35-fold increase of the cueR/cueA/cueA-cueR is not visible.

2. The addition of Cu(I) appears to have only a small effect on CueR binding to the cueA promoter and statistical analyses of these data should be performed to reveal the significance of the data. It would be helpful to indicate the molar concentration of the labeled PcueA fragment instead of µg, otherwise it is difficult to estimate the stoichiometry of the PcueA-CueR complex.

3. Considering that CueR has an exceptionally high Cu(I) affinity (10-16 - 10-21 M), the authors need to indicate which measures they took to really ensure Cu-free conditions. It would also be helpful, if the they would monitor the basal Cu-concentrations in their `Cu-free´ conditions and upon BCS-treatment.

4. The authors state that deleting the putative Cu-chaperone CueZ has no effect on Cu-tolerance. These data should be shown and it should also be indicated whether the genome-wide search revealed any other putative Cu chaperones. This is important, because CopA-like ATPases generally receive their Cu(I) via Cu-chaperones

5. The colonization experiments indicate that CueR potentially regulates additional genes besides cueA. In this respect, it would be important to mention that CueR in P. aeroginosa regulates in addition to copA1, also the production of the two copper chaperones CopZ1 and CopZ2, the RND-type multidrug efflux pump MexPQ-OpmE and several hypothetical proteins that are linked to glycolysis and fatty acid metabolism (summarized in Hofmann et al., 2021). Thus, these targets could be tested to identify other genes that are regulated by CueR in P. chlororaphis and influence root colonization.

The presence of additional CueR targets, such as CopZ (CueZ), could also explain why upon copper stress, the double mutant shows a much stronger reduction in CFU/ml than the single mutants.

6. The authors discuss in detail the potential binding mode of CueR to the promoter region and how this is affected by Cu(I) binding. Previous studies have reported distinct conformational changes in CueR depending on the promoter to which it is

bound. However, as the present study does not experimentally investigate CueR conformational changes or DNA bending, this section could be shortened.

7. I am not convinced that the observed regulation is indeed `completely different from the regulation mode in E. coli´ (line 347/348), because the effect of Cu(I) on CueR binding in the EMSA test is not very pronounced.

8. The statement that at low Cu-concentrations inactive CueR binds to the promoter region (line 383 ff), somehow contradicts the early statement that `binding ability is significantly enhanced upon copper ion addition´(line 347/348). The effect of Cu binding could be tested by using a cysteine-mutant of CueR.

9. The authors propose a dual function model for CueR, i.e., that it regulates copper homeostasis and regulatory targets of colonization. Without identifying these additional targets, the authors cannot exclude that other player of the copper homeostasis network also influence colonization. In particular when considering that generally the number of CueR targets in bacteria is very low (2 in E. coli and 5 in P. aeruginosa).

Before addressing the specific comments, we would like to express our heartfelt gratitude to both reviewers for their professional feedback on our manuscript. Your insights have been immensely helpful in improving the quality of our manuscript and refining our future research logic. We truly appreciate your time and dedicated efforts. Below is our point-by-point response to the reviewers' comments, with each comment followed by our detailed reply. Should you have any remaining questions or concerns regarding our responses, please feel free to contact us. We are fully prepared to make further efforts to enhance the quality of this manuscript.

Reviewer #1:

This manuscript investigates the regulatory mechanism of CueR on copper homeostasis in *Pseudomonas chlororaphis* zm-1, a plant biocontrol bacterium. The authors identify that *cueR* and *cueA* form a *cueRA* operon, which is positively regulated by CueR in the presence of copper. This work provides insights into the diversity of copper homeostasis regulation in beneficial plant-associated bacteria. However, I have several concerns about the work.

Q1. The greenhouse pot experiments show that copper homeostasis imbalance impairs colonization in peanut rhizospheres. However, the current data mainly focuses on population counts. Did the authors observe any phenotypic changes in the peanut plants themselves when inoculated with the Δ*cueR* or Δ*cueA* mutants under copper stress compared to the wild type?

Response: First of all, we would like to express our special thanks for your meticulous consideration of our experiment. In the experiment, we observed no significant differences in growth between peanuts under copper stress and those grown normally. There were also no distinct differences in growth among the copper-stressed peanuts inoculated with different bacterial strains. The copper concentration we selected was at the lower end of soil copper pollution levels to prevent high concentrations from causing peanut mortality. Regarding the selection of experimental materials, we chose peanuts of largely uniform size for germination, and then selected those with basically identical root lengths to soak in the bacterial suspension before conducting the pot experiment. During the experiment, we used a graduated cylinder to measure the watering volume to avoid human error causing differences in peanut growth. To ensure the accuracy of the experimental results, we replanted the peanuts after receiving the reviewer's comments and measured their plant heights after 14 days for statistical analysis. The statistical results (Fig. 6C) indicated that there were no significant differences in peanut growth.

Q2. The study utilizes EMSA to verify that Cu^+ enhances the binding of CueR to the *cueA* promoter region. While this demonstrates direct regulation, the binding affinity (K_d) is not explicitly calculated or discussed. Have the authors performed quantitative analyses, such as ITC or MST, to determine the binding affinity of CueR to the promoter in the presence versus the absence of Cu^+?

Response: Thank you for providing this methodological strategy; we indeed overlooked this critical point. Consequently, we re-purified the CueR protein and amplified the promoter fragment via PCR. Our university has a Nano ITC（Waters/TA，USA） instrument capable of performing this experiment, and we booked a session to use it. However, the instrument technician informed us that the measurement requirements stipulate a concentration of 10 μM to several hundred μM for the sample cell (titrand), and several hundred μM to tens of mM for the syringe (titrant). Generally, the titrant concentration is required to be at least 10 times higher than that of the titrand.

According to these requirements, if we use CueR as the titrand (the purified CueR protein concentration was determined to be 1 mg/mL, i.e., 60 μM, using the BCA assay), the *cueA* promoter fragment would need to reach a concentration of at least 600 μM. However, the DNA fragment cannot reach such a high concentration; once its concentration reaches 65 μM, it hits the anisotropy critical point and forms liquid crystals.

Conversely, if we place the DNA fragment in the sample cell as the titrand, the experimental protocol requires rinsing the sample cell at least twice, with a minimum sample volume of 1300 μL per rinse, totaling 2600 μL. To obtain a large quantity of the promoter fragment, we simultaneously amplified 30 tubes of 50 μL samples. After gel electrophoresis and gel extraction, we recovered 120 μL of the pure fragment at a measured concentration of 102 ng/μL (i.e., 0.5 μM). If we were to obtain the minimum required concentration of 10 μM DNA fragment for the experiment, we would need to

amplify at least 13,000 tubes of samples, which is impractical for us. Therefore, we could not obtain the required 2600 μL of 10 μM DNA fragment sample.

Nevertheless, we still attempted the experiment by loading the 60 μM CueR protein into the sample cell and titrating it with the 0.5 μM promoter fragment. Unsurprisingly, we were unable to obtain a normal titration curve; the data points were scattered and could not be fitted to determine the binding affinity (K_d) between the CueR protein and the *cueA* promoter fragment.

Despite this outcome, we are deeply grateful for the method you suggested, which helped us further refine and evaluate our experimental results.

Q3.In Figure 2A, the labeling is insufficiently clear; for instance, it is difficult to distinguish which strain is represented by the orange line. Furthermore, the growth curves for the different strains are heavily intertwined, making them hard to read. For example, in the presence of 0.3 mM Cu^{2+}, the growth curve for the wild-type (WT) strain is completely obscured. I suggest improving the visual presentation, perhaps by separating the panels or using distinct markers to ensure all data points are visible.

Response: Indeed, as you suggested, the restoration of copper tolerance in the gene complementation strain led to the overlapping phenomenon in the growth curves. To improve this, we plan to remove the growth curves of the gene complementation strains. After discussion, we have confirmed that their removal does not affect the readability of the experimental conclusions, while also facilitating readers' understanding.

Q4. I recommend redrawing the proposed model in Figure 7. The current illustration fails to clearly depict the mechanism by which Cu^{+} enhances the binding affinity of CueR to the *cueA* promoter, as demonstrated in your EMSA results. Additionally, the figure should explicitly illustrate the biological significance of CueR regulating *cueA* to provide a more comprehensive summary of your findings. Specifically, how this interaction facilitates copper efflux and maintains homeostasis.

Response: We are deeply grateful for your careful review of our manuscript. After further discussion, we agree that the original presentation was indeed somewhat inappropriate. In the model proposed in our manuscript, we demonstrated the following mechanism: under normal intracellular copper levels, the *cueRA* operon is transcribed at a basal level, producing copper-free CueR. At this stage, the copper-free CueR binds as a dimer to the promoter region of the *cueRA* operon but remains inactive, it does not distort the DNA. In contrast, when intracellular copper levels are excessively high, copper-bound CueR binds to the promoter region of the *cueRA* operon as a dimer, distorting the DNA and promoting high-level transcription of the *cueRA* operon, resulting in the production of a large amount of CueA to expel copper ions.

Furthermore, we agree that the biological significance of CueR regulating *cueA* should be explicitly demonstrated. Maintaining this copper homeostasis is crucial for *Pseudomonas chlororaphis* zm-1 to adapt to environmental copper stress, assisting its stable colonization of peanut roots and ultimately enhancing the peanut's resistance to pathogens. Therefore, we have further supplemented the manuscript by illustrating the

colonization process of *P. chlororaphis* zm-1 on peanut roots, explicitly highlighting its connection to copper homeostasis.

Q5. Line 85, "soft" should be "soft".

Response: We are somewhat confused by this comment and do not fully understand its intent. If you have any further suggestions or clarification on this point, please let us know so that we can make the necessary revisions.

First of all, we would like to express our sincere gratitude for taking the time out of your busy schedule to review our manuscript, and we truly appreciate your hard work and dedication. We found your comments to be exceptionally professional, and they will be of great help in guiding our revisions and further improving the quality of our paper.

Prior to providing the specific comments, you suggested that we cite the report regarding the *cueRA* operon in *Pseudomonas putida* (Adaikkalam & Swarup, 2002). We fully agree with your suggestion and are willing to tone down our claims regarding novelty. However, we would like to clarify that this is indeed the first report of this mechanism in *Pseudomonas chlororaphis*, and that our study specifically involves the bacterial colonization ability in the peanut rhizosphere.

In the following sections, we provide a point-by-point response to your specific comments.

Reviewer #2:

Wang et al report on the role of the transcriptional regulator CueR in regulating copper homeostasis in *Pseudomonas chlororaphis* zm-1.

CueR belongs to the MerR family of transcriptional regulators and has been characterized in detail in *Escherichia coli* and *Pseudomonas aeruginosa*, whereas comparatively less is known about CueR in plant growth-promoting bacteria. Here, the authors identify the *cueAR* operon in *Pseudomonas chlororaphis* zm-1 and characterize its role in copper homeostasis. A similar operon organization has previously been

reported in *Pseudomonas putida* (Adaikkalam & Swarup, 2002), and this study should therefore be cited in the relevant section. In light of these earlier findings, the novelty claims of the present manuscript should also be toned down accordingly.

Overall, this is a solid study that demonstrates that CueR in *Pseudomonas chlororaphis* zm-1 is an important regulator of copper homeostasis, primarily via regulating the expression of the CopA-like copper-exporting ATPase CueA. In contrast, the predicted role of *cueAR* in rhizosphere colonization is more speculative.

For improving the quality of the manuscript, the following issues should be addressed:

Q1. The more than 80-fold increase of *cueA* expression is not visible in Fig. 3B, this needs to be explained. This also applies to Fig. 5B, where the about 35-fold increase of the *cueR*/*cueA*/*cueA*-*cueR* is not visible.

Response: First of all, we are deeply grateful to you for pointing out our mistake; this was indeed an oversight in our work. When we performed the first gel electrophoresis on the qRT-PCR products, we realized that the excessive loading volume resulted in overly thick and visually unappealing bands. Therefore, when re-running the gel, we chose to dilute the samples before loading, which led to the results presented in our initial submission. However, we overlooked the inherent variations between samples, which was an oversight on our part. Consequently, we chose to uniformly dilute the remaining samples and perform the gel electrophoresis again. Interestingly, we noticed that there was no significant difference in band intensity between the 0.1 mM Cu^{2+} and 0.2 mM Cu^{2+} treatment groups, a result that initially surprised us. However, after consulting relevant materials, we concluded that this result is scientifically accurate. In

qRT-PCR, while different samples require a different number of cycles to reach the threshold, the program is set for 40 cycles. Ultimately, all samples enter the plateau phase, exhausting the enzymes and dNTPs in the reaction system and causing the reaction to terminate. This explains why samples with different threshold cycles (C_t values) end up yielding almost identical band intensities on the gel. We have now updated the figures to present the results of this re-run gel.

Q2. The addition of Cu(I) appears to have only a small effect on CueR binding to the *cueA* promoter and statistical analyses of these data should be performed to reveal the significance of the data. It would be helpful to indicate the molar concentration of the labeled *PcueA* fragment instead of µg, otherwise it is difficult to estimate the stoichiometry of the *PcueA*-CueR complex.

Response: To better facilitate the understanding and estimation of the stoichiometry of the *PcueA*-CueR complex, we calculated the amount of the added *PcueA* DNA fragment, and the result was approximately 0.08 µM（Marked-Up Manuscript，line 211）. We have re-annotated the EMSA results.

Q3. Considering that CueR has an exceptionally high Cu(I) affinity (10^{-16}-10^{-21} M), the authors need to indicate which measures they took to really ensure Cu-free conditions. It would also be helpful, if the they would monitor the basal Cu-concentrations in their Cu-free conditions and upon BCS-treatment.

Response: To ensure a copper-free environment, the deionized water used in our experiments was Milli-Q water that had undergone additional purification. Furthermore, a 1 mM concentration of the metal ion chelator EDTA was added during both protein dialysis and GST-tag cleavage processes. To further determine the specific copper levels, we measured the basal copper concentration using ICP-OES, which was found to be 0.002 mg/L. To evaluate the residual copper levels after adding BCS in the EMSA experiments, we attempted detection using the BCA reagent; unfortunately, due to the extremely low copper concentration, it fell below the detection limit. Moreover, because ICP-OES only detects total copper levels rather than free copper ions, we were unable to utilize that method for this specific measurement either.

Q4. The authors state that deleting the putative Cu-chaperone CueZ has no effect on Cu-tolerance. These data should be shown and it should also be indicated whether the genome-wide search revealed any other putative Cu chaperones. This is important, because CopA-like ATPases generally receive their Cu(I) via Cu-chaperones

Response: In the early stage of our experiment, to search for the presence of copper chaperones in *P. chlororaphis* zm-1, we used BLAST to query the amino acid sequences of CopZ1 and CopZ2 reported in *Pseudomonas aeruginosa*, as well as CueZ reported in *Pseudomonas fluorescens* SBW25, against the *P. chlororaphis* zm-1 proteome. The retrieval results are summarized in the table below.

qseqid	sseqid	pident	length	mismatch	gapopen	qstart	qend	sstart	send	evalue	bitscore

copZ1	WP_007923288.1	36.066	61	39	0	1	61	1	61	1.49e-10	48.9
CopZ2	WP_007923288.1	66.154	65	22	0	1	65	1	65	1.65e-26	89.0
CueZ	WP_007923288.1	78.462	65	14	0	1	65	1	65	3.23e-35	111

The results indicated that all queried copper chaperones pointed to the protein numbered WP_007923288.1. Given that it shared the highest similarity with CueZ, and based on spatial structural modeling using SWISS-MODEL which confirmed its structural similarity to CueZ, we designated it as CueZ.

Next, we constructed a *cueZ* knockout strain (Δ*cueZ*) to determine its tolerance to copper ions. In *P. aeruginosa*, CopZ2 primarily functions as a copper sink to help the bacteria mitigate the toxicity of free Cu^+ , and the knockout of *copZ*2 leads to a decrease in bacterial copper tolerance. However, in *P. chlororaphis* zm-1, the deletion of *cueZ* had almost no effect on its copper tolerance, which was observed through both growth curves (Fig. S6A). Nevertheless, after subjecting the strains to 0.2 mM $CuSO_4\cdot 5H_2O$ copper stress for 2 hours, we measured the intracellular copper levels and found that the intracellular copper concentration of the Δ*cueZ* strain decreased from 0.390 ± 0.027 in the wild type to 0.269 ± 0.03 (Fig. S6B). This indicates that CueZ does function as a copper sink in *P. chlororaphis* zm-1. The modification results are in lines 433-448 of the Marked-Up Manuscript.

Q5. The colonization experiments indicate that CueR potentially regulates additional genes besides *cueA*. In this respect, it would be important to mention that CueR in *P. aeroginosa* regulates in addition to *copA*1, also the production of the two copper chaperones CopZ1 and CopZ2, the RND-type multidrug efflux pump MexPQ-OpmE and several hypothetical proteins that are linked to glycolysis and fatty acid metabolism (summarized in Hofmann et al., 2021). Thus, these targets could be tested to identify other genes that are regulated by CueR in *P. chlororaphis* and influence root colonization. The presence of additional CueR targets, such as CopZ (CueZ), could also explain why upon copper stress, the double mutant shows a much stronger reduction in CFU/ml than the single mutants.

Response: In the literature regarding copper homeostasis in *Pseudomonas aeruginosa* (Novoa-Aponte, Ramírez et al. 2019, Hofmann, Hirsch et al. 2021), it has been reported that CueR possesses at least four additional binding sites across the genome. These sites are located upstream of the *cusABC* gene cluster, *copZ*1, the PA3515-3519 gene cluster, and *copZ*2, respectively. Therefore, we performed a search on the *Pseudomonas aeruginosa* PAO1 genome and downloaded the amino acid sequences of the proteins encoded by these genes. Subsequently, we used BLAST to query these sequences against the *P. chlororaphis* zm-1 protein database.

No similar proteins were retrieved for PA3518 and PA3519. Although searching the *Pseudomonas aeruginosa* PA3522 amino acid sequence against the *Pseudomonas chlororaphis* zm-1 proteome yielded numerous candidates, analysis of the promoter regions upstream of these gene clusters revealed a lack of the palindromic sequences

required for CueR binding. Given that the CueR-binding motif has been reported to be highly conserved, we ruled out the possibility of its regulatory involvement. This indicates a substantial genomic divergence between *Pseudomonas aeruginosa* and *Pseudomonas chlororaphis*. Consequently, we were unable to investigate the underlying mechanisms by referencing existing literature.

**qseqid**	**sseqid**	**pident**	**length**	**mismatch**	**gapopen**	**qstart**	**qend**	**sstart**	**send**	**evalue**	**bitscore**
PA3515	WP_124311698.1	36.648	352	216	4	7	356	1	347	7.88e-69	217
PA3515	WP_238399350.1	26.000	100	71	1	175	274	46	142	5.02e-06	45.1
PA3516	WP_162094543.1	34.174	436	275	4	53	482	20	449	3.70e-63	209
PA3516	WP_007929192.1	26.887	212	120	8	132	324	115	310	1.03e-09	57.8
PA3516	WP_009051440.1	27.536	207	118	5	134	325	108	297	2.61e-06	47.0
PA3517	WP_162094543.1	36.111	432	270	3	15	442	14	443	9.70e-74	237
PA3517	WP_007929192.1	29.442	197	126	5	99	287	114	305	4.67e-17	80.9
PA3517	WP_009051440.1	27.103	214	153	3	96	308	102	313	3.82e-09	55.8

PA3517	WP_124335329.1	25.532	235	145	7	100	315	130	353	4.90e-08	52.4
PA3517	WP_007928510.1	27.468	233	149	8	97	311	126	356	2.22e-06	47.4
PA3522	WP_162096183.1	55.587	1047	449	6	1	1036	1	1042	0	1093
PA3522	WP_162095524.1	54.598	1044	462	6	1	1036	1	1040	0	1071
PA3522	WP_162094883.1	49.188	1047	523	4	1	1040	1	1045	0	970
PA3522	WP_081362490.1	38.752	1058	626	12	5	1052	3	1048	0	726
PA3522	WP_162095477.1	42.703	1021	565	9	4	1017	2	1009	0	721
PA3522	WP_124340183.1	40.561	1033	602	8	5	1033	3	1027	0	720
PA3522	WP_124309411.1	34.787	1032	641	14	1	1030	1	1002	2.98e-170	524
PA3522	WP_162095208.1	30.472	1060	685	19	1	1044	1	1024	3.43e-132	424
PA3522	WP_162096045.1	31.256	1043	673	19	6	1036	10	1020	6.66e-132	423
PA3522	WP_124338266.1	32.083	1066	646	20	7	1042	7	1024	1.79e-131	422
PA3522	WP_162096093.1	24.36	1055	741	19	5	1027	7	1036	8.37e-66	238
PA3522	WP_124308187.1	23.58	1039	694	24	15	1019	20	992	3.15E-51	193

PA3522	WP_041985754.1	23.238	1050	715	24	15	1027	17	1012	4.35e-47	180
PA3522	WP_124307412.1	22.321	1008	710	19	8	990	16	975	5.29e-46	177
PA3522	WP_162095002.1	28.629	496	338	7	10	501	11	494	2.72e-44	172
PA3522	WP_162095002.1	28.571	322	201	9	726	1031	749	1057	1.58e-21	98.6
PA3522	WP_028682268.1	27.16	162	115	2	344	505	624	782	1.51e-07	52.8
PA3523	WP_009049255.1	42.973	370	204	6	2	367	4	370	3.35e-91	278
PA3523	WP_028682908.1	39.831	354	199	6	12	358	15	361	5.57e-76	238
PA3523	WP_237509764.1	40.173	346	199	4	30	369	41	384	2.09e-72	229
PA3523	WP_162095525.1	42.47	332	182	4	42	366	49	378	1.65e-68	219
PA3523	WP_162095476.1	35.849	371	220	7	25	382	19	384	1.80e-49	169
PA3523	WP_009047413.1	31.88	367	219	9	9	363	10	357	1.22e-47	164

PA3523	WP_124299473.1	32.432	370	243	6	2	365	3	371	9.01e-43	151
PA3523	WP_162095207.1	33.233	331	217	3	29	358	65	392	9.47e-40	144
PA3523	WP_162096044.1	31.287	342	219	8	27	358	28	363	8.02e-38	137
PA3523	WP_162095980.1	26.629	353	213	8	34	365	41	368	1.13e-19	87
PA3523	WP_081360710.1	26.761	355	219	13	30	353	28	372	2.90e-14	70.9
PA3523	WP_162095766.1	26.923	338	199	12	55	353	54	382	3.56e-13	67.8
PA3523	WP_162095001.1	27.333	300	204	6	57	352	73	362	3.27e-11	61.6
PA3523	WP_124307740.1	24.011	354	255	5	3	356	2	341	5.19e-11	60.8
PA3523	WP_038583155.1	31.373	153	100	3	63	213	48	197	1.08e-10	59.3
PA3523	WP_162096094.1	26.733	303	183	10	67	355	122	399	1.42e-10	59.7
PA3523	WP_124307367.1	28.328	293	137	9	67	300	49	327	1.22e-09	56.6

PA3523	WP_162094227.1	28.061	196	133	3	8	200	3	193	4.96e-08	51.6
PA3523	WP_009050395.1	26.174	149	106	3	67	213	48	194	5.25e-08	51.2
PA3523	WP_162095463.1	29.224	219	107	7	63	248	46	249	6.80e-08	51.2
PA3523	WP_162094226.1	24.654	361	247	7	13	365	12	355	6.81e-08	51.2
PA3523	WP_124334641.1	32.624	141	79	3	49	189	52	176	1.01e-07	50.4
PA3523	WP_124299088.1	26.146	371	161	16	25	293	14	373	1.97e-07	50.1
PA3523	WP_162094452.1	30.556	144	96	3	69	211	50	190	3.00e-06	45.8

Q6. The authors discuss in detail the potential binding mode of CueR to the promoter region and how this is affected by Cu(I) binding. Previous studies have reported distinct conformational changes in CueR depending on the promoter to which it is bound. However, as the present study does not experimentally investigate CueR conformational changes or DNA bending, this section could be shortened.

Response: We have discussed the potential binding modes between CueR and the promoter region in detail. However, due to limitations in our experimental conditions

and technical capabilities, we are unable to further investigate these conformational changes in the DNA and protein. We readily accept your suggestion and will shorten this section accordingly. At the same time, we have revised Fig. 7 to reduce the description of DNA structural deformation. The modification results are in lines 329-336 of the Marked-Up Manuscript.

Q7. I am not convinced that the observed regulation is indeed `completely different from the regulation mode in *E. coli* (line 347/348), because the effect of Cu(I) on CueR binding in the EMSA test is not very pronounced.

Response: Our experimental results indeed do not fully demonstrate a "completely distinct regulatory mode from that in *Escherichia coli*." We are willing to modify this statement to state that our findings are inconsistent with previous conclusions, thereby providing a different reference value for understanding the regulatory mechanisms of CueR. The modification results are in line 354 of the Marked-Up Manuscript.

Q8. The statement that at low Cu-concentrations inactive CueR binds to the promoter region (line 383 ff), somehow contradicts the early statement that `binding ability is significantly enhanced upon copper ion addition´(line 347/348). The effect of Cu binding could be tested by using a cysteine-mutant of CueR.

Response: Regarding the contradiction you pointed out, we would like to explain the reasoning behind our conclusion. Through EMSA experiments, we found that CueR can directly bind the *cueA* promoter fragment without the addition of exogenous Cu^{+}.

When exogenous Cu^+ was added, CueR at the same concentration bound a greater amount of the *cueA* promoter fragment, as evidenced by the higher intensity of the bound DNA bands. Our original phrasing may have lacked sufficient rigor; therefore, we are willing to change"under low copper concentrations, inactive CueR binds to the promoter region" to "when CueR is not bound to Cu^+, inactive CueR binds to the promoter region". The modification results are in lines 394 of the Marked-Up Manuscript.

To prove that inactive CueR can bind to the promoter region when it is not bound to Cu^+, we mutated the CueR-Cu^+ binding sites at C112 and C121, changing the cysteine residues to serine (TGC to TCC, and TGT to TCT). We then induced and purified this mutant protein. The subsequent EMSA experiments demonstrated that this mutation did not affect the binding ability of CueR to the *cueA* promoter fragment. Furthermore, the addition of Cu^+ did not alter the binding capacity between this mutant CueR and the *cueA* promoter fragment (Fig. 4C).

Q9. The authors propose a dual function model for CueR, i.e., that it regulates copper homeostasis and regulatory targets of colonization. Without identifying these additional targets, the authors cannot exclude that other player of the copper homeostasis network also influence colonization. In particular when considering that generally the number of CueR targets in bacteria is very low (2 in *E. coli* and 5 in *P. aeruginosa*).

Response: We appreciate your insightful suggestion. We agree that CueR may indeed possess other potential binding sites in *P. chlororaphis* zm-1. Given that bacterial

colonization is a complex, multifactorial process, our experimental results suggest that CueR may be indirectly involved in this process. As our strain serves as a biocontrol agent against *Sclerotium rolfsii*, stable colonization of the peanut rhizosphere is a prerequisite for its biocontrol efficacy, which is why we have prioritized the study of the colonization process. Based on the analysis provided in our previous response, we are currently unable to definitively identify the specific potential binding sites of CueR. Therefore, we will tentatively introduce the concept of a dual function model in the Discussion section of the manuscript. Elucidating the underlying mechanisms of this model will be a focus of our future research.

Re: Spectrum03981-25R1 (The transcriptional regulator CueR positively regulates the expression of the *cueRA* operon to maintain copper homeostasis in *Pseudomonas chlororaphis* zm-1)

Dear Dr. Gang Wang:

Your manuscript has been accepted, and I am forwarding it to the ASM production staff for publication. Your paper will first be checked to make sure all elements meet the technical requirements. ASM staff will contact you if anything needs to be revised before copyediting and production can begin. Otherwise, you will be notified when your proofs are ready to be viewed.

Data Availability: ASM policy requires that data be available to the public upon online posting of the article, so please verify all links to sequence records, if present, and make sure that each number retrieves the full record of the data. If a new accession number is not linked or a link is broken, provide production staff with the correct URL for the record. If the accession numbers for new data are not publicly accessible before the expected online posting of the article, publication may be delayed; please contact ASM production staff immediately with the expected release date.

Publication Fees: For information on publication fees and which article types have charges, please visit our website. We have partnered with Copyright Clearance Center (CCC) to collect author charges. If fees apply to your paper, you will receive a message from no-reply@copyright.com with further instructions. For questions related to paying charges through RightsLink, please contact CCC at ASM_Support@copyright.com or toll free at +1-877-622-5543. CCC makes every attempt to respond to all emails within 24 hours.

ASM Membership: Corresponding authors may join or renew ASM membership to obtain discounts on publication fees. Need to upgrade your membership level? Please contact Customer Service at Service@asmusa.org.

Embargo Policy: A press release may be issued as soon as the manuscript is posted on the Spectrum Latest Articles webpage. The corresponding author will receive an email with the subject line "ASM Journals Author Services Notification" when the article is available online.

Cover Image Submissions: If you would like to submit a potential Cover Image, please email a file and a short legend to spectrum@asmusa.org. Please note that we can only consider images that (i) the authors created or own and (ii) have not been previously published. By submitting, you agree that the image can be used under the same terms as the published article. Image File requirements: TIF/EPS, 7.5 inches wide by 8.25 inches tall (at least 2,250 pixels wide by 2,475 pixels tall), minimum 300 dpi resolution (600 dpi preferred), RGB, and no figure elements, e.g., arrows or panel labels. The legend should be a short description of the image, 1-2 sentences recommended. Please use the ASM Journals Cover Image Sizing Tool (https://journals.asm.org/pb-assets/html/asm-cover-sizing-tool.html) to ensure that your proposed cover image meets our size requirements.

The ASM Journals program strives for constant improvement in our submission and publication process. Please tell us how we can improve your experience by taking this quick Author Survey.

Thank you for submitting your paper to Spectrum.

Sincerely,
Katharina Kujala
Editor
Microbiology Spectrum

Reviewer #1 (Comments for the Author):

Thank you for including the comments in the manuscript. The manuscript is now much improved.

Reviewer #2 (Comments for the Author):

The authors have addressed all my previous concerns.
