# Supplementary material for: The transcriptional regulator CueR positively regulates the expression of the cueRA operon to maintain copper homeostasis in Pseudomonas chlororaphis zm-1
Source: Microbiol Spectr. 2026 Sep 17;14(10):e03981-25. doi: 10.1128/spectrum.03981-25 (PMC13688801; doi:10.1128/spectrum.03981-25)
Supplement: Supplementary file 2 [file spectrum.03981-25-s0001.pdf]

Supporting Information for

The transcriptional regulator CueR positively regulates the expression of the *cueRA* operon to maintain copper homeostasis in *Pseudomonas chlororaphis* zm-1

Zhongkang Wang,[a] Chen Li,[a] Zhenming Lei,[a] Sijia Duan,[a] Fengying Liu,[a,b] Shaowei Wang,[a,b]# Gang Wang,[a,b]#

Running Head: CueR-Regulated Copper Homeostasis in *P. chlororaphis*

[a]School of Life Sciences, Henan University, Kaifeng 475004, China

[b]Engineering Research Center for Applied Microbiology of Henan Province, Kaifeng 475004, China

#Address correspondence to Shaowei Wang, wangsw69@henu.edu.cn

#Address correspondence to Gang Wang, gwangbio@henu.edu.cn.

This file includes:

Figuers S1-S6

Tables S1-S2

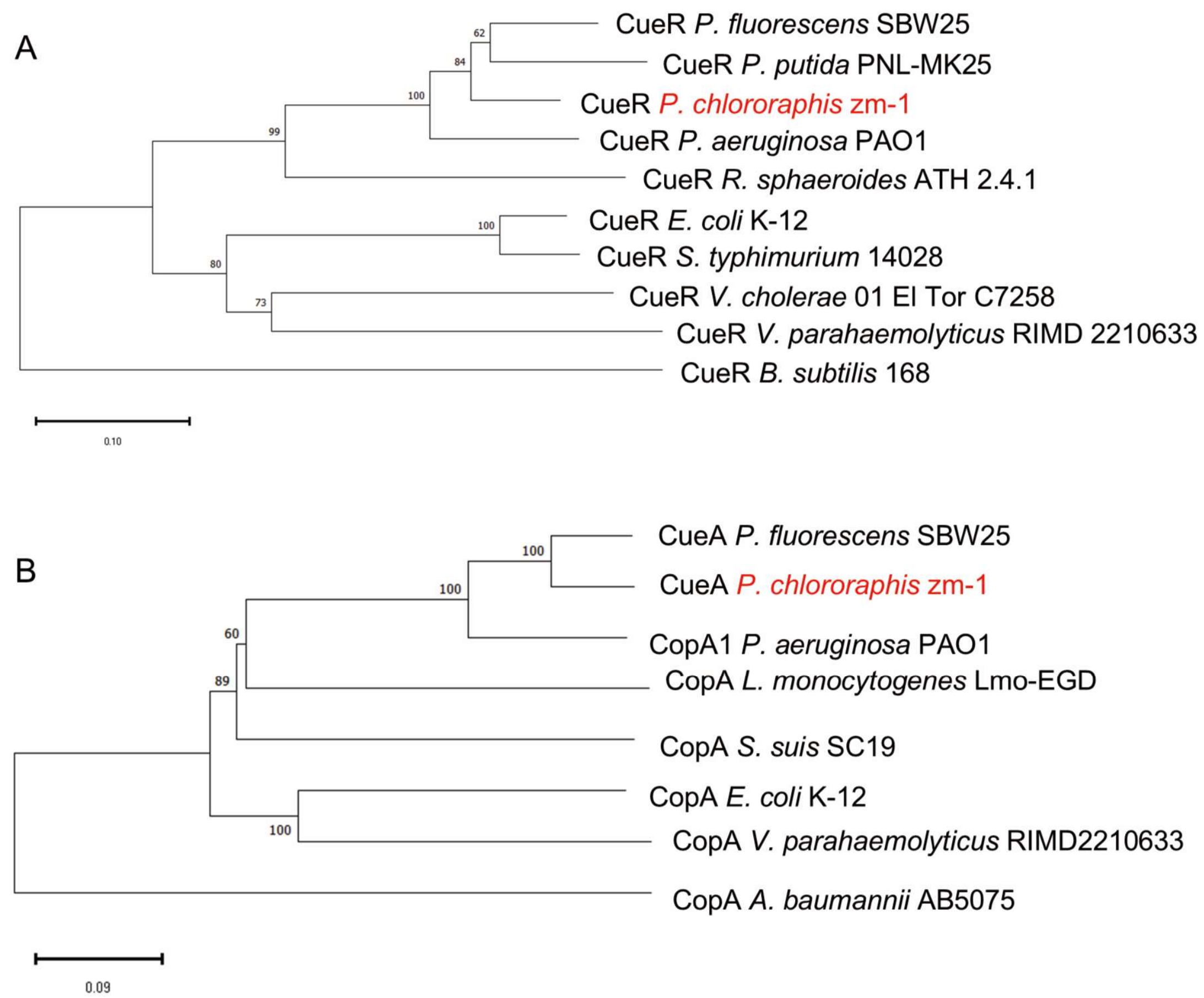

Fig. S1. Construction of phylogenetic trees for CueR and CueA homologs. (A) Phylogenetic tree of CueR from *P. chlororaphis* zm-1 and homologous sequences from other species. (B) Phylogenetic tree of CueA from *P. chlororaphis* zm-1 and homologous P_{1B}-type ATPase sequences from other species.

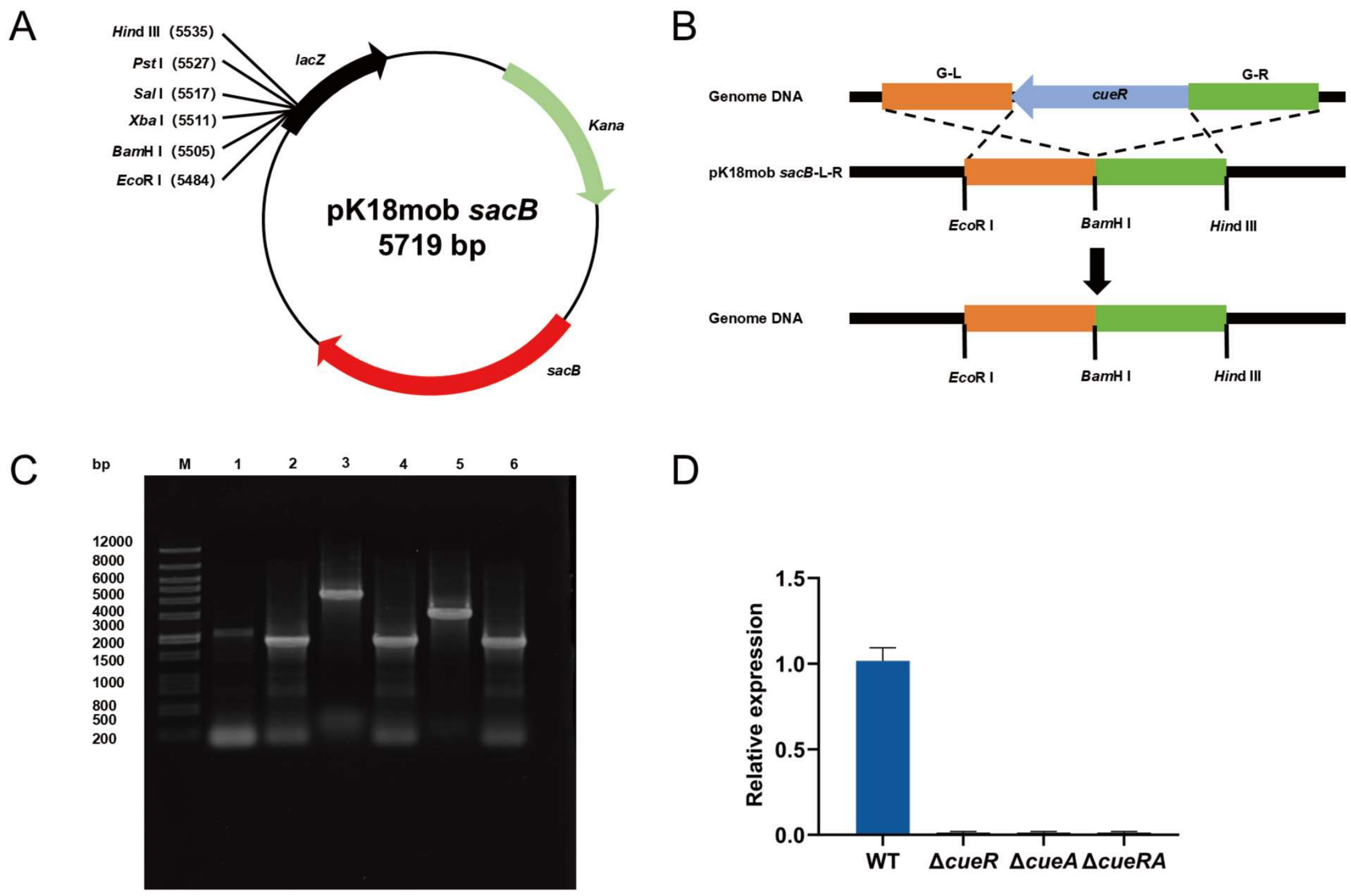

Fig. S2. Validation of mutant strains. (A) Map of the pK18mob *sacB* shuttle vector used for gene deletion in *P. chlororaphis* zm-1. (B) Schematic of the mutant strategy. (C) PCR verification of gene deletions. Lanes 1, 3, 5: WT control; lane 2: *cueR* deletion; lane 4: *cueA* deletion; lane 6: *cueRA* deletion. M: DNA marker (Beyotime, Shanghai). (D) Expression levels of target genes in deletion strains relative to the WT. Data are represented as the mean ± SD (n = 3).

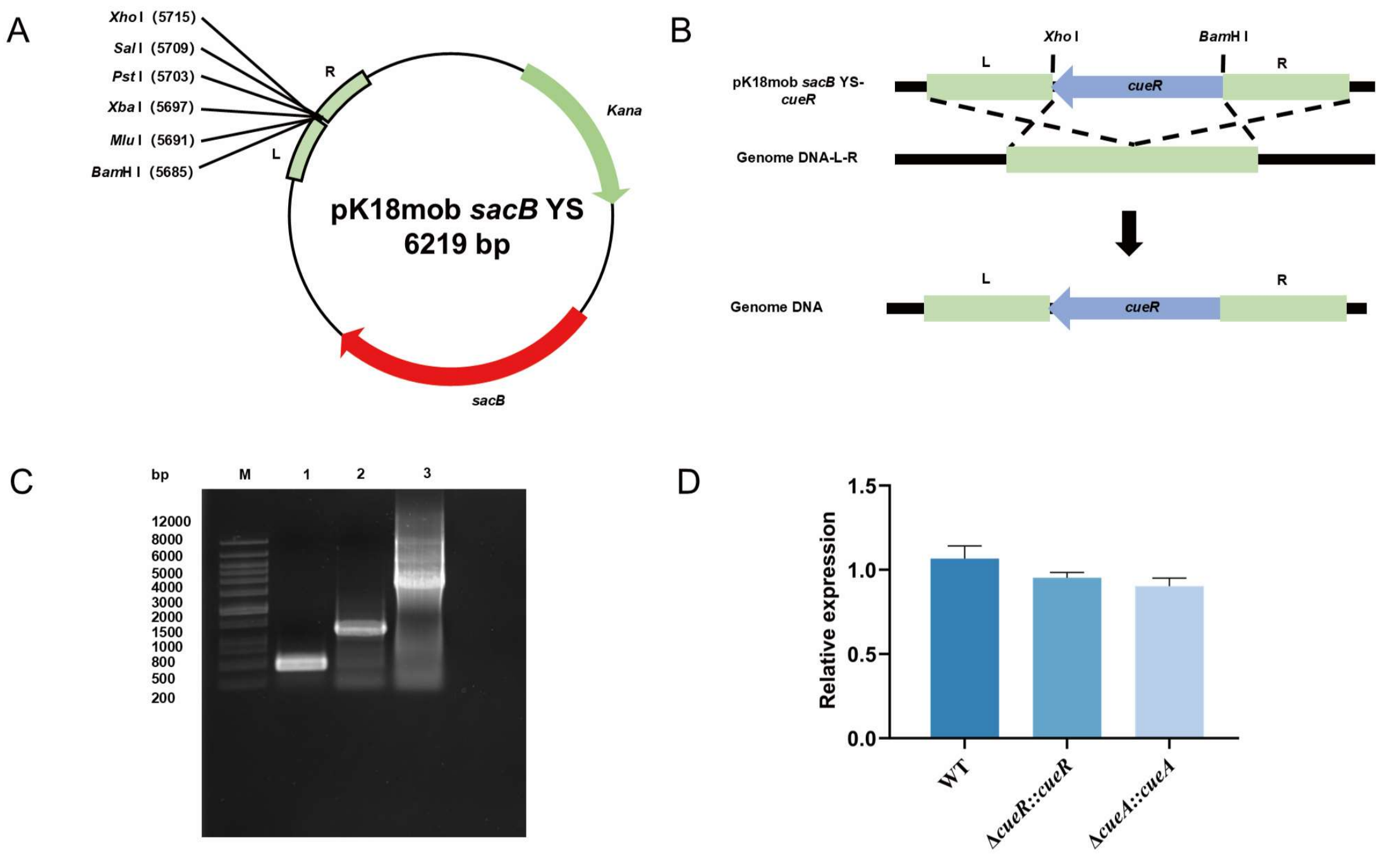

Fig. S3. Validation of complementation strains. (A) Map of the pK18mob *sacB* YS shuttle vector used for gene complementation. (B) Schematic of the gene complementation strategy. (C) PCR verification of gene complementation. Lane 1: WT control; lane 2: *cueR* complementation strain; lane 3: *cueA* complementation strain. M: DNA marker (Beyotime, Shanghai). (D) Expression levels of target genes in complementation strains relative to the WT. Data are represented as the mean ± SD (n = 3).

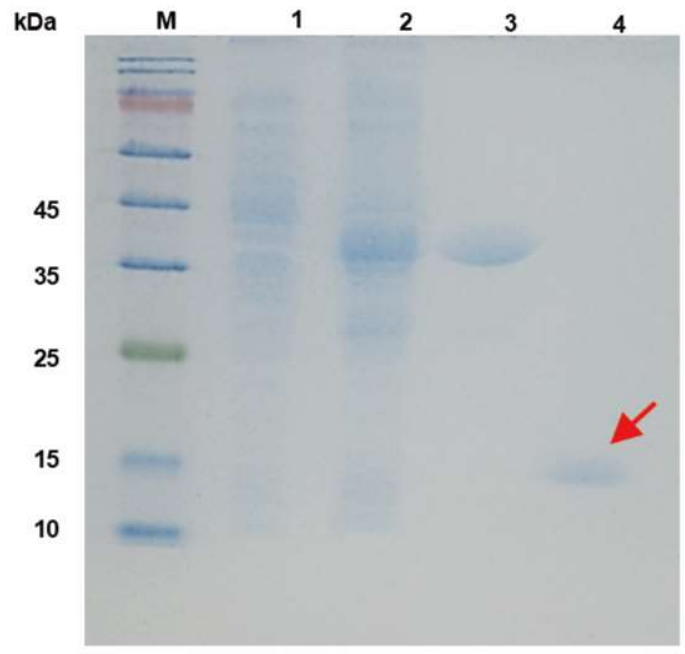

Fig. S4. SDS-PAGE analysis of CueR purification. Lane M: protein molecular weight marker (Thermo Fisher, USA). Lane 1: uninduced control. Lane 2: protein expression after induction with 0.1 mM IPTG. Lane 3: GST-tagged CueR purified by GST-tag gravity column. Lane 4: tag-free CueR protein after PreScission protease (Beyotime, China) treatment and purification.

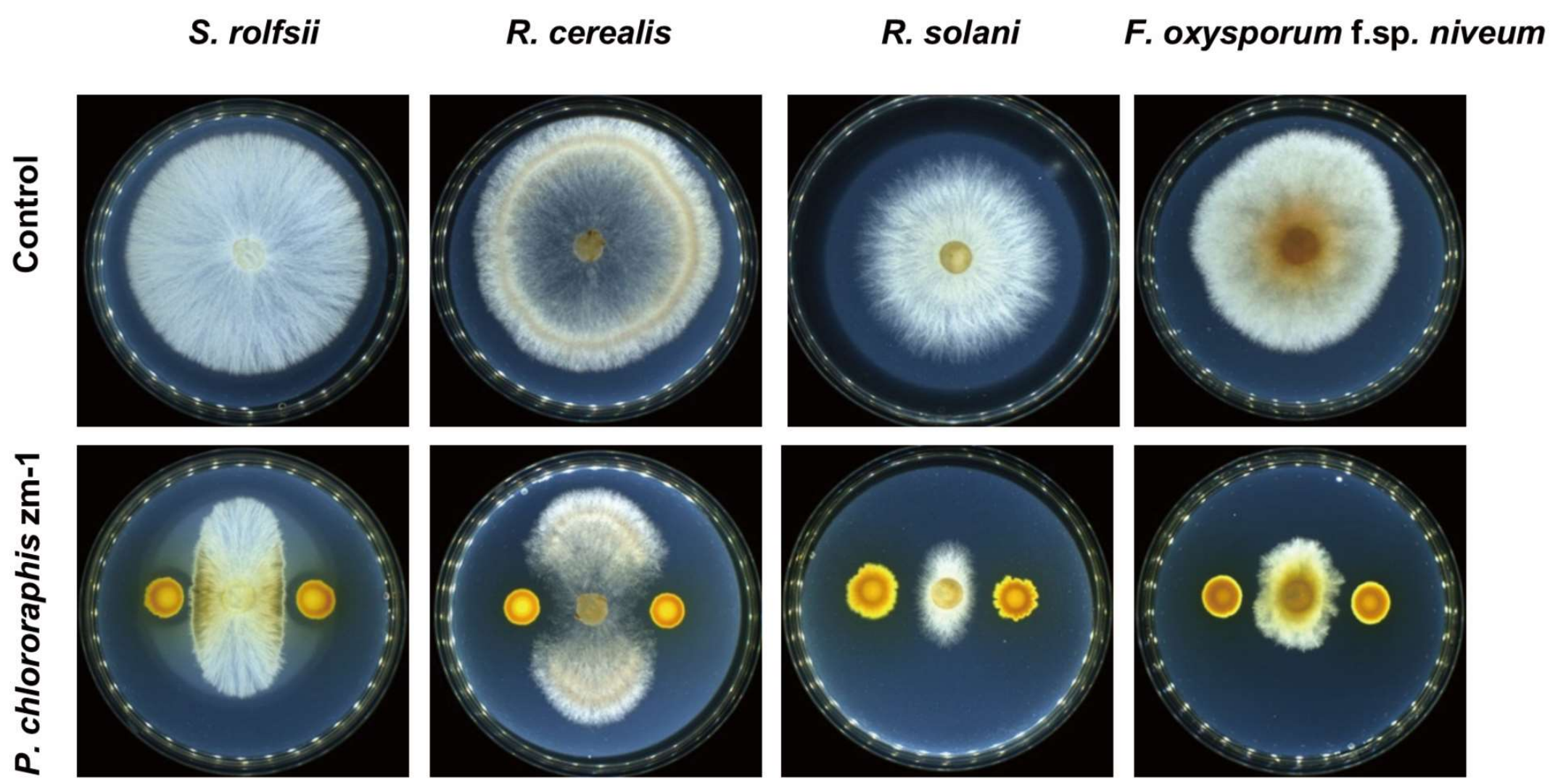

Fig. S5. Antagonistic assay of *P. chlororaphis* zm-1 against phytopathogenic fungi on PDA agar plates. A fungal mycelial plug was placed at the center of an agar plate, with *P. chlororaphis* zm-1 inoculated 2 cm away. After 4 days of static incubation at 25°C, the plates were observed and photographed using a Scan 1200 imaging system (Interscience, France). This assay evaluated the antagonistic activity of strain zm-1 against *Sclerotium rolfsii*, *Rhizoctonia cerealis*, *Rhizoctonia solani*, and *Fusarium oxysporum* f. sp. *niveum*.

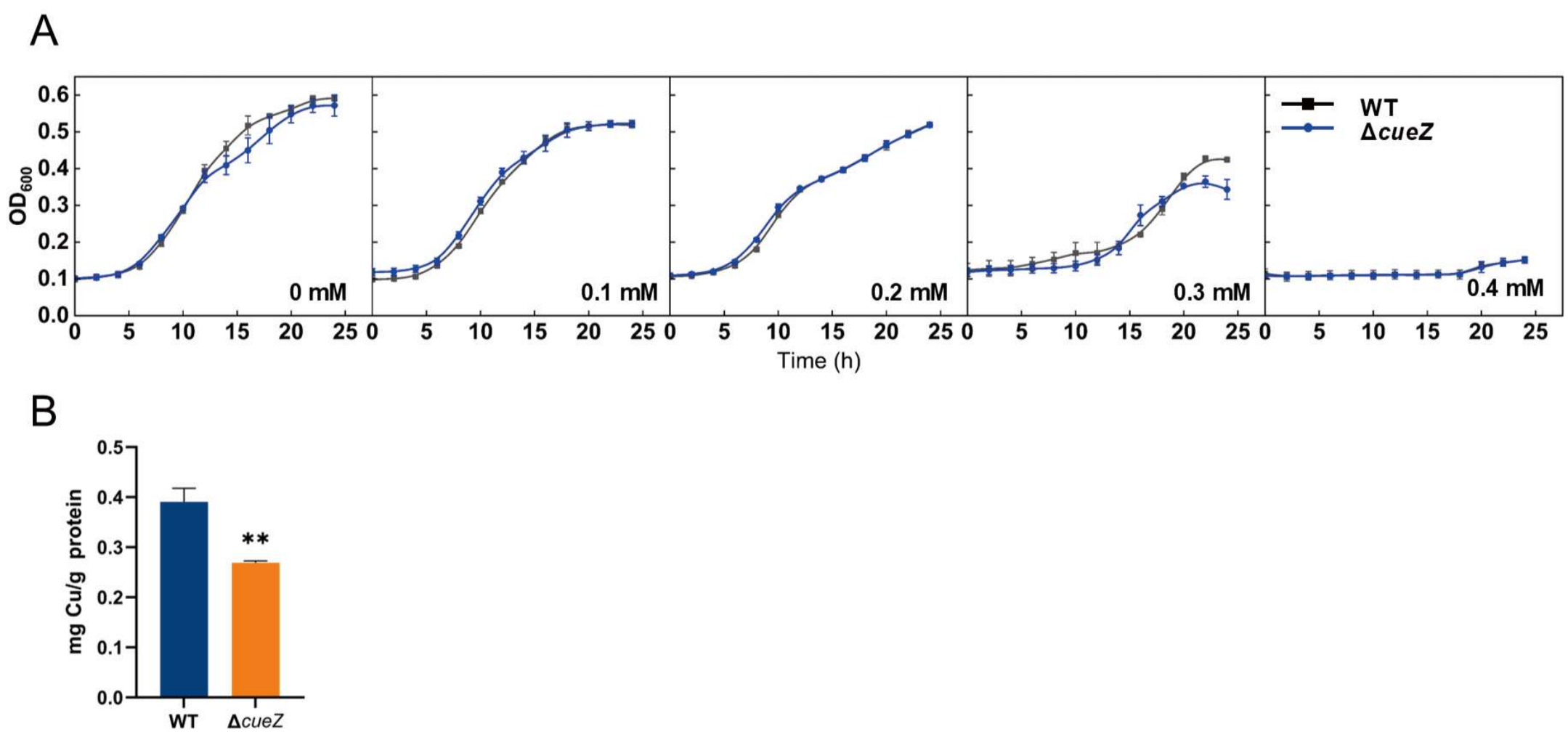

Fig. S6. Determination of Δ*cueZ* growth curve and intracellular copper concentration. (A) Growth curves of mutant strains in LB medium supplemented with 0-0.4 mM $CuSO_4 \cdot 5H_2O$. (B) Intracellular copper content measured by atomic absorption spectroscopy. Intracellular copper levels in exponential-phase cells after 30 min exposure to 0.2 mM $CuSO_4 \cdot 5H_2O$ in LB medium. Data are expressed as mean ± SD (n = 3). Two-tailed unpaired Student's *t*-test were used to calculate *P* values. ** $P < 0.01$.

Table S1. Strains and plasmids used in this study.

Strains	Genotype or description	Source
***P. chlororaphis* zm-1 Strains**		
zm-1	Wild type strain in this study, Cm^+	Laboratory Storage, Isolated from *A. asphodeloides* root.
Δ*cueR*	The *cueR* gene deletion mutant strain, Cm^+	Construct in this study
Δ*cueA*	The *cueA* gene deletion mutant strain, Cm^+	Construct in this study
Δ*cueRA*	The double deletion strain of *cueR* and *cueA* genes, Cm^+	Construct in this study
Δ*cueZ*	The *cueZ* gene deletion mutant strain, Cm^+	Construct in this study
Δ*cueR*::*cueR*	Δ*cueR* supplemented with *cueR* gene by allelic exchange, Cm^+	Construct in this study
Δ*cueA*::*cueA*	Δ*cueA* supplemented with *cueA* gene by allelic exchange, Cm^+	Construct in this study
***E. coli* Strains**		
116 (*pir*+)	Plasmid propagation	Purchased from BioVector NTCC
S17-1(*λpir*)	Bacterial conjugation	Purchased from BioVector NTCC
BL21 (DE3)	Protein expression, F^- *ompT* $hsdS_B$ ($r_B^- m_B^-$) *gal dcm lacY*1 (DE3)	Purchased from BioVector NTCC
**Plasmids**		
pK18mob *sacB*	For gene deletion, Kan^+	Takara, Dalian
pGEX-6p-1	Protein expression vector, Amp^+	Takara, Dalian
pK18mob *sacB*YS	For reverse complementation, derived from pK18mob *sacB*, Kan^+	Stored in our laboratory
cueA-pK18mob *sacB*	For gene deletion, Kan^+	Construct in this study
cueR-pK18mob *sacB*	For gene deletion, Kan^+	Construct in this study
cueRA-pK18mob *sacB*	For gene deletion, Kan^+	Construct in this study
cueZ-pK18mob *sacB*	For gene deletion, Kan^+	Construct in this study
P*cueRA*+*cueA* -pK18mob *sacB*YS	For reverse complementation, Kan^+	Construct in this study
P*cueRA*+*cueR*-pK18mob *sacB*YS	For reverse complementation, Kan^+	Construct in this study
cueR-pGEX-6p-1	Protein expression vector, Amp^+	Construct in this study

Table S2. Primers used in this study (5'-3').

Primer Name	Sequence 5′-3′	Description
cueA-up-*Eco*RI-s	ACACACGAATTCCGGCAACATGGCGTCCTACTTC	For *cueA* gene deletion
cueA-up-*Xho*I-a	CACACTCGAGCTACTGTTGAAAACCTGGAAAC	For *cueA* gene deletion
cueA-down-*Xho*I-s	ACACCTCGAGCATGCCGGCAATCGGCAGGTCA	For *cueA* gene deletion
cueA-down-*Xba*I-a	CACACATCTAGAGATCACCTCGTCGGCGGACAAG	For *cueA* gene deletion
cueA-*Xba*I-s	CTAGTCTAGAGGCGACGTACTGAAGTTCCG	For *cueA* gene complementatio
cueA-*Bam*HI-a	CACACTCGAGTCATCGGGCGTCCTCCAGGT	For *cueA* gene complementation
cueR-up-*Eco*RI-s	ACACACGAATTCCAAGGTGCGCAACAGCCGTCAG	For *cueR* gene deletion
cueR-up-*Bam*HI-a	CACACAGGATCCGCGGGTGCTGCGCGTGATGTAG	For *cueR* gene deletion
cueR-down-*Bam*HI-s	ACACGGATCCCCGATGTTCATCGGGCGTCCTC	For *cueR* gene deletion
cueR -down-*Hin*dIII-a	CACAAAGCTTGACCACCTGCTGCAATTGGCTG	For *cueR* gene deletion
cueZ-up-*Xba*I-s	ACACTCTAGATGTAGCCGGCATCCTGC	For *cueZ* gene deletion
cueZ-up-*Xho*I-a	CACACTCGAGGACGCTTCTCCTGTAGTG	For *cueZ* gene deletion
cueZ-down-*Xho*I-s	ACACCTCGAGGCGGATTTAACCGGTCTT	For *cueZ* gene deletion
cueZ -down-*Hin*dIII-a	CACAAAGCTTACCGTGCATTGATCTGCG	For *cueZ* gene deletion
cueR -*Bam*HI-s	ACACACGGATCCATGAACATCGGCCAAGCAGCCCGCA	For *cueR* gene complementation
cueR -*Xho*I-a	CACACACTCGAGTTACGCGCAGCACCCGCCCGAAG	For *cueR* gene complementation
yzcx-S	GCACTTGGATTTACGCCGTCGGTAG	For gene complementation verify
yzcx-A	GTCGGTTTCGTTGGACTGATCGACA	For gene complementation verify
cueR-RT PCR-s	ATCGGACAATCCGGGCGGT	For *cueR* expression measurement
cueR-RT PCR-a	TCGCGGGACCTGGGGTTTT	For *cueR* expression measurement
cueA-RT PCR-s	AGGTTGTAGACGAAGGCC	For *cueA* expression measurement
cueA-RT PCR-a	ATATCGGCATTGCCATGG	For *cueA* expression measurement
cueRA-RT PCR-s	CCATGGCGCTGTCCAGCGT	For *cueRA* expression measurement
cueRA-RT PCR-a	TACAGGCGGTAGCCGCTGT	For *cueRA* expression measurement
rpoD-RT PCR-s	AGAGTGCTCCGGATGCGGA	For *rpoD* expression measurement
rpoD-RT PCR-a	GCGATCGCGCCCATCACTT	For *rpoD* expression measurement

Note: Sequences which marked with "___" stand for restriction enzyme cutting site.
